# Anisotropic Müller glial scaffolding supports a multiplex lattice mosaic of photoreceptors in zebrafish retina

**DOI:** 10.1186/s13064-017-0096-z

**Published:** 2017-11-15

**Authors:** Mikiko Nagashima, Jeremy Hadidjojo, Linda K. Barthel, David K. Lubensky, Pamela A. Raymond

**Affiliations:** 1 Department of Molecular, Cellular, and Developmental Biology, University of Michigan, 830 North University Avenue, Ann Arbor, MI 48109-1048 USA; 2 Department of Physics, University of Michigan, 450 Church Street, Ann Arbor, MI 48109-1040 USA; 3 Microscopy and Image Analysis Laboratory, University of Michigan, Ann Arbor, MI USA

**Keywords:** Retina, Müller glia, Photoreceptor mosaic, Mechanical anisotropy, Spatial patterning, Laser ablation

## Abstract

**Background:**

The multiplex, lattice mosaic of cone photoreceptors in the adult fish retina is a compelling example of a highly ordered epithelial cell pattern, with single cell width rows and columns of cones and precisely defined neighbor relationships among different cone types. Cellular mechanisms patterning this multiplex mosaic are not understood. Physical models can provide new insights into fundamental mechanisms of biological patterning. In earlier work, we developed a mathematical model of photoreceptor cell packing in the zebrafish retina, which predicted that anisotropic mechanical tension in the retinal epithelium orients planar polarized adhesive interfaces to align the columns as cone photoreceptors are generated at the retinal margin during post-embryonic growth.

**Methods:**

With cell-specific fluorescent reporters and in vivo imaging of the growing retinal margin in transparent juvenile zebrafish we provide the first view of how cell packing, spatial arrangement, and cell identity are coordinated to build the lattice mosaic. With targeted laser ablation we probed the tissue mechanics of the retinal epithelium.

**Results:**

Within the lattice mosaic, planar polarized Crumbs adhesion proteins pack cones into a single cell width column; between columns, N-cadherin-mediated adherens junctions stabilize Müller glial apical processes. The concentration of activated pMyosin II at these punctate adherens junctions suggests that these glial bands are under tension, forming a physical barrier between cone columns and contributing to mechanical stress anisotropies in the epithelial sheet. Unexpectedly, we discovered that the appearance of such parallel bands of Müller glial apical processes precedes the packing of cones into single cell width columns, hinting at a possible role for glia in the initial organization of the lattice mosaic. Targeted laser ablation of Müller glia directly demonstrates that these glial processes support anisotropic mechanical tension in the planar dimension of the retinal epithelium.

**Conclusions:**

These findings uncovered a novel structural feature of Müller glia associated with alignment of photoreceptors into a lattice mosaic in the zebrafish retina. This is the first demonstration, to our knowledge, of planar, anisotropic mechanical forces mediated by glial cells.

**Electronic supplementary material:**

The online version of this article (10.1186/s13064-017-0096-z) contains supplementary material, which is available to authorized users.

## Background

Modular architecture is a defining characteristic of the nervous system with its precise spatial segregation and regular arrangement of cellular components and circuits. The vertebrate retina is an exceptionally clear example of this organizational property [1]. Most retinal neuron types are arranged in evenly spaced planar arrays (mosaics), which ‘tile’ the retinal surface [2, 3]. The mechanisms that pattern homotypic mosaics in the retina include tangential cell dispersion in response to repulsive, local, cell-cell interactions among like-cell types and, in some cases, selective cell death, resulting in the creation of an ‘exclusion zone’ around individual neurons [4]. Surprisingly, mosaics of different neuronal types in the retina are independent and uncorrelated [5, 6]. Uniquely in teleost fish, spectral subtypes of cones are arranged into lattice mosaics in which the positions of different cone subtypes are precisely correlated [7]. These multiplex, lattice mosaics, in which the spatial positions of several distinct cell types are correlated, exhibit a diversity of patterns across fish species and are a specific teleost feature not seen in cone photoreceptors in other vertebrate retinas, e.g., birds [6] or primates [8, 9]. Clearly, homotypic patterning mechanisms are insufficient to explain multiplex, lattice mosaics of teleost cones. However, we currently have very limited knowledge about the organizational strategies that build the lattice mosaic of teleost photoreceptors and not even the most basic information about how cell packing, spatial arrangement, and cell identity are coordinated.

**Fig. 1:**
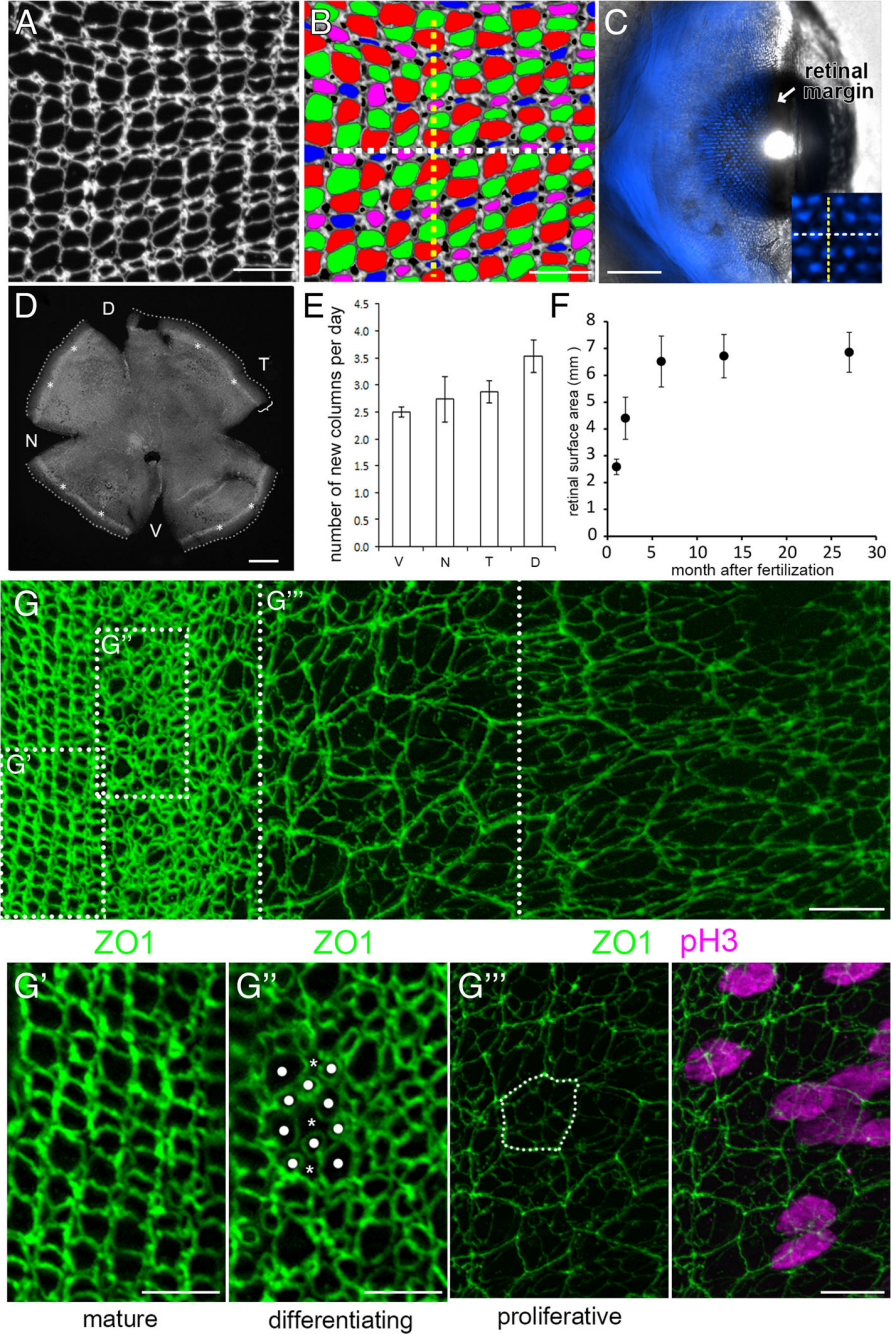
Emergence of the photoreceptor lattice mosaic in rapidly growing juvenile zebrafish. (A) Retinal flat-mount immunolabeled for ZO1. (B) Same as panel A, with cone profiles colored per spectral identity (UV cones in magenta); rods in black. Cone columns (dashed yellow line); rows of UV-Blue cones (dashed white line). (C) Live imaging, early larval *Tg(sws2*:mCherry*; ruby)* zebrafish eye, Blue cone-specific reporter (blue) viewed from the dorsal side, cornea to the right; spherical lens. Inset: Blue cone rows (white dashed line) orthogonal to, and columns (yellow dashed line) parallel to, the retinal margin. (D) Flat-mount retina; ring of EdU-labeled cells (asterisks) parallel to the retinal margin; cells added in the 10-day interval after the EdU pulse (bracket). D,Dorsal; N, Nasal; T, Temporal; V, Ventral. (E) Columns of cones added per day by retinal quadrant. Mean ± 1 s.d., *n* = 3. (F) Retinal surface area as a function of age. Mean ± 1 s.d., *n* = 4–6 for each age. (G) Flat-mount preparation of retinal margin and overlying retinal pigmented epithelium (RPE) in a juvenile zebrafish (maximum intensity *z-*projection of ZO1, in green). (See also Additional file 2: Figure S1 and Additional file 3: Movie S1.) (G’-G”) High magnification of boxes in G. Mitotic marker, pH3 (magenta; overlay in G”’, right panel). In the differentiating zone, immature photoreceptors have rounded profiles (dots) and Müller glial apical profiles are irregular polygonal shapes (asterisks). RPE cells (dashed line) in a separate, closely apposed epithelium (see Additional file 2: Figure S1B and Additional file 3: Movie S1). Scale bars: 10 μm (A, B, G, G”’); 100 μm (C); 200 μm (E); 5 μm (G’, G”)

In adult zebrafish four spectrally and morphologically distinct cone photoreceptor subtypes organize at the apical retinal surface into a lattice mosaic of single cell width rows and columns of cones, with rod photoreceptors preferentially inserted between cone columns (Fig. 1A and B) [10]. The majority of cells in adult fish retina are generated by progenitors located in a germinal zone at the retinal margin [11, 12], and new cones organize into single cell width columns parallel to the growing margin [10, 13]. In earlier work we argued that the arrangement of cones into straight, single-file columns in the lattice mosaic must reflect an underlying anisotropy in mechanical stresses within the retinal sheet, with a large tensile stress parallel to the columns and much weaker forces in the orthogonal direction [10, 14]. Current methods for measuring mechanical forces in intact tissues remain limited [15, 16], but with laser ablation the mechanical force supported by an element before its ablation can be estimated from the recoil of surrounding cells after ablation [17–19]. This approach has been successfully used to determine the degree of stress anisotropy in epithelial sheets in vivo [20–22]. We adapted this method to probe the role of glial cells in supporting the ordered, columnar cone cell packing in zebrafish retina.

Here we explored the temporal sequence of photoreceptor lattice mosaic formation, providing the first in vivo view of how the multiplex mosaic is created. Importantly, the organization of photoreceptors into a planar lattice mosaic is a property only of cells generated post-embryonically in the zebrafish retina, whereas photoreceptors generated in the embryonic and early larval retina are not arranged in a lattice mosaic [10, 11]. (In zebrafish, embryonic development lasts until ~72 h post-fertilization, larval stages are up to ~ 4 weeks post-fertilization, and juvenile stages up to ~3 months [23].) Thus, the standard techniques for live imaging and time lapse recording that have been used to great advantage to study retinal development in zebrafish embryos and early larvae, including analysis of cell lineage, retinal lamination, and formation of neural circuits [24–26], are not directly applicable to investigating formation of the lattice mosaic pattern. We instead developed methods for non-invasive live imaging and targeted cell ablation in the growing retinal margin of semi-transparent juvenile zebrafish (1–2 months old), which lack iridophore and melanin pigments. Our results show that spatial positioning of nascent Red and UV cones occurs prior both to cell packing into linear columns and to planar polarized distribution of Crumbs (Crb)-mediated homophilic adhesion [27], but roughly coincident with the first appearance of parallel lines of glial cell processes that presage the cone columns. Laser ablation of Müller glia near the retinal margin in living fish provided evidence of the predicted anisotropic tension in the retinal epithelium [10]. We propose that a network of glial apical processes that are linked by N-cadherin junctional contacts [28, 29] sustains the anisotropic mechanical forces that align differentiating cone photoreceptors. The important contribution of retinal Müller glia to structural support and lamination of the retina is well-established [30, 31], and the viscoelastic properties of the radial fibers of Müller glia have been measured [32–35]. In contrast, this study reveals an unexpected biomechanical property of Müller glia that is operative in the planar epithelial dimension of the retina (orthogonal to the radial fibers) and likely mediates alignment of the lattice mosaic of photoreceptors.

## Methods

### Zebrafish

Fish were maintained at ~28° C on a 14/10 h light/ dark cycle with standard husbandry procedures [36]. Zebrafish lines included wild-type TL, *Tg(trβ2:* tdTomato*)* and *Tg(crx:*mCFP*)* [25], *Tg(−3.7rho:*EGFP*)kj2* [37]*, Tg(−5.5sws1*: EGFP*)kj9* [38], *Tg(−3.2sws2*: mCherry*)mi2007* [10], and *Tg(gfap:*EGFP*)mi2002* [39]. For live imaging experiments, transgenic lines were crossed into ‘*ruby’* [40], carrying *albino(slc45a2)* ^*b4/b4*^ *and roy* ^*a9/a9*^ pigment mutations (Zebrafish International Resource Center, Eugene, OR).

### Histology

For retinal flat mounts, adult zebrafish were placed in the dark for 3 h prior to dissection. After euthanizing by rapid chilling/hypothermia and cervical transection, the eye was enucleated and a small hole was made ventrally at the choroid fissure. With microscissors, the ventral hole was extended along the radial axis of the eyeball for orientation. The lens was removed, the eyecup was placed in phosphate buffered saline (PBS), and the neural retina was gently removed from the pigmented retinal epithelium with forceps, except at the peripheral retinal margin, where the overlying pigmented retinal epithelium was retained to preserve the retinal germinal zone. Short relaxation cuts were made along the perimeter, and the retina was fixed in 4% paraformaldehyde with 5% sucrose in 0.1 M phosphate buffer (PB), pH 7.4, at 4° C overnight.

Flat-mount retinal immunocytochemistry was performed essentially as previously described [10], with the addition of antigen retrieval. After fixation, retinas were treated with 10 mM sodium citrate in 0.05% Tween 20 (pH 6.0) in boiling water for 5 min, and then removed from the heating plate and allowed to cool in the hot water for 5 min. After rinsing in PBS with 0.5% Triton X-100, free-floating retinas were incubated in blocking buffer (10% normal goat serum, 1% Tween 20, 1% Triton X-100, 1% DMSO in PBS, pH 7.4, with 0.1% sodium azide) for 2 h. Primary and secondary antibodies were diluted in PBS with 0.5% normal goat serum, 1% Tween 20, 1% Triton X-100, 1% DMSO in PBS (pH 7.4) with 0.1% sodium azide, and the incubations performed at room temperature overnight. Tissues were washed in the washing buffer, and the retinas were mounted on microscope slides with Prolong Gold or Prolong Diamond (Invitrogen, Carlsbad, CA) with the photoreceptor side down. Antibodies used in this study are listed in Additional file 1: Table S1.

For retinal cross sections, the cornea was punctured and the eyeball was fixed intact in 4% paraformaldehyde with 5% sucrose in PB, pH 7.4, at 4° C overnight. After rinsing with 5% sucrose in PBS, the lens was carefully removed, and the tissue processed for cryosectioning as previously described [41]. The tdTomato signal was enhanced by immunostaining with anti-dsRed as previously described [42]. Nuclei were stained with Hoechst 33342 (Invitrogen, Carlsbad, CA) prior to mounting in Prolong Gold or Prolong Diamond (Invitrogen, Carlsbad, CA).

### Microscopy and image analysis of fixed specimens

Retinal preparations (flat-mount or cryosections) were imaged with a Zeiss AxioImage ZI Epifluorescent Microscope (Carl Zeiss Microimaging, Thornwood, NY) equipped with an ApoTome attachment for optical sectioning using structured illumination, and processed with Adobe Photoshop CS6 Extended (Adobe Systems, San Jose, CA). The Leica Application Suite X (Leica Microsystems, Wetzlar, Germany), Image J (https://imagej.nih.gov/ij/) or Imaris 8.3.0 (Bitplane, Zurich, Switzerland) software packages were used for image analysis and movie production.

### Analysis of post-embryonic retinal growth

To label proliferating progenitor cells in the retinal margin, juvenile fish (1-month-old) were allowed to swim in a solution of 125 μM 5-ethynyl-2’-deoxyuridine (EdU) in aquarium system water for 4 h, then returned to normal water for 10 days prior to retinal dissection. After 2 h of fixation in 4% paraformaldehyde with 5% sucrose in 0.1 M PB, pH 7.4, at room temperature, retinas were treated with sodium citrate for antigen retrieval, as described above. Retinas were then rinsed with 3% bovine serum albumin (BSA) in PBS/ 1% Tween 20/ 1% Triton X-100 for 5 min twice. EdU detection was performed with the Click-iT™ Plus EdU Alexa Fluor™ 488 Imaging Kit (Invitrogen, Carlsbad, CA). After washing in PBS/ 1% Triton X-100/ 1% Tween 20, retinas were placed in a blocking solution for ZO1 flat mount immunocytochemistry. Retinal surface area was extracted from low magnification images of flat-mount retinal preparations with Image J, (https://imagej.nih.gov/ij/), using the automated threshold and surface area functions in the Analyze tool.

### Cell counts

Optical section *z*-stack images were obtained from the margins of flat-mounted retinas of transgenic *Tg(trβ2*:tdTomato*)* fish immunostained with antibody to ZO1. Cell profiles at the level of the OLM were obtained by selectively projecting the ZO1 channel using a MATLAB (https://www.mathworks.com) program developed in-house (available on request). The program reconstructs the OLM surface three-dimensionally by averaging the ZO1 signal with a Gaussian kernel in the *z*-direction and interpolating in the *x*- and *y*-directions. This reconstructed surface was then used to selectively capture the tdTomato signals at the level of the OLM. The cell profiles from the ZO1 channel were segmented using a watershed algorithm with manually-placed seeds. Irregularly-shaped Müller glial profiles were excluded by hand at this stage. Average tdTomato intensity at the level of the OLM for each segmented profile was then calculated and clustered into *trβ2*:tdTomato positive and negative groups using a *k*-means algorithm [43].

### Müller glia ridge analysis

Profiles of GFP-labeled Müller glial processes at the level of ZO1-immunolabel from the retinal margin in *Tg(gfap:* EGFP) flat-mount preparations were captured with the MATLAB routine developed for cell counts. We used an ImageJ plugin (https://imagej.nih.gov/ij/) for ridge detection with anisotropic steerable filters [44] to define ridges and to compare their orientation *θ* to the direction *θ*_*c*_ of the cone columns. Briefly, for each pixel in the original image, the filter returns a value indicating how anisotropic (i.e., ridge-like) the intensity field in the vicinity of the pixel is and an angle corresponding to the optimal ridge orientation. The “Refine features” function of the plugin then suppresses pixels with low ridge-like scores and isolated pixels with higher scores that do not form part of continuous ridge lines [44]. We found that a 4th order filter with width of 8 pixels (corresponding to the typical thickness of GFP signals between columns) best discriminates the thick, between-column expanded apical glial profiles from the thinner, within column lamellae, based on the least number of false positives. The former give mostly long, straight ridge lines parallel to the column, whereas the latter tend to appear as many short, curved ridge lines. To eliminate most of the latter and keep only the former, the ridge angles *θ* and intensities returned by the filter were imported into MATLAB, where they were compared to the direction *θ*_*c*_ of the columns, defined by applying a Fast Fourier Transform to the unfiltered image. (The direction with the largest Fourier amplitude is perpendicular to the columns.) Pixels with deviation |*θ–θ*_*c*_| more than 30° from the direction of the columns are deemed spurious and were eliminated. To further filter the result, ridges less than 20 pixels long were also removed. (If the same procedure is applied, but keeping pixels with angles near a value other than the correct column direction *θ*_*c*_, few or no long ridges remain; thus, long, straight Müller glial ridges can be detected only parallel to the columns, as expected.)

### Live imaging

To visualize steps in formation of the photoreceptor lattice mosaic in situ, we developed methods for live imaging of the growing retinal margin in larval and juvenile zebrafish up to ~2-months-old. Due to the anatomy of the head and eye, the dorsal retina was the most accessible for analysis. Growth rates of individual zebrafish during larval and juvenile stages show extreme variability, and therefore size, not age, is a better proxy for developmental stage [23].

In the transparent *ruby* genetic background, fluorescent reporters labeling retinal cells can be visualized in living fish. Stereomicroscopic images of a 3-week-old zebrafish (0.72 cm standard body length, excluding the tail) were obtained with a Zeiss AxioImage ZI Epifluorescent Microscope (Carl Zeiss Microimaging, Thornwood, NY). The fish was anesthetized with 0.336 mg/ml Tricaine S/MS-222 (Western Chemical Inc., Ferndale, WA) and mounted dorsal side down on a No. 1.5 coverslip with 1% low-melting agarose in aquarium system water. The fish mounted on the coverslip was inverted onto concave microscope slide and the dorsal retina was imaged with a Zeiss EC Plan-NEOFLUAR, 0.3 NA, 10X objective lens, with both brightfield (differential interference contrast, DIC) and epifluorescent optics.

For confocal microscopic imaging, juvenile zebrafish 0.74 to 1.19 cm standard body length were anesthetized with 0.336 mg/ml Tricaine S/MS-222 (Western Chemical Inc., Ferndale, WA). When opercular movements ceased, the fish was placed in a 50 mm glass bottom Petri dish with a No. 1.5 coverslip (MatTek Corporation, Ashland MA) and oriented dorsal side down, tipped laterally so that the dorsal aspect of one eye was directly apposed to the coverslip. Kimwipe tissues (Sigma-Aldrich, St. Louis, MO) moistened with anesthetic water were used to stabilize the fish during imaging. The fish remained anesthetized in the imaging chamber for up to 1 h in the absence of gill ventilation and perfusion. Continued viability was confirmed by the presence of a heart beat and blood flow to peripheral tissues, including the eye.

Confocal *z*-stack images were captured with a Leica TCS SP8 LSCM (Leica Microsystems, Wetzlar, Germany) equipped with a tunable Chameleon 2-photon Ti:Sapphire laser (Coherent, Santa Clara, CA) and Leica 40X PL APO CS2 Water Immersion lens, 1.1 NA with 650 μm working distance. The Multiphoton (MP) laser was tuned to 800 nm and Leica HyD hybrid detectors tuned to 450–500 nm for Cyan; 500–550 nm for EGFP; 576–650 nm for tdTomato. The Argon laser (Excitation: 488 nm Emission: HyD 524–561 nm for EGFP; Excitation: 514 nm Emission: HyD 577–745 nm for tdTomato) was also used for capturing images of the germinal zone and far peripheral retinal margin, but laser penetration was insufficient for capturing signals from more central retina. For post-acquisition processing, *z-*stacks were loaded into the Leica Application Suite X (Leica Microsystems, Wetzlar, Germany), Image J (https://imagej.nih.gov/ij/) or Imaris 8.3.0 (Bitplane, Zurich, Switzerland) software for volume rendering and maximum intensity *z-*projection movies.

### Müller glial ablation and analysis of epithelial tension

Juvenile transgenic zebrafish with a Müller glia reporter (*gfap:*EGFP) were anesthetized with TricaineS/MS-222 (0.336 mg/ml) and oriented with the dorsal side of one eye apposed to a No.1.5 coverslip in a 50 mm glass bottom Petri dish. Pre-ablation and post-ablation MP images were acquired at 400 Hz acquisition speed with a resolution of 512 × 512 pixels in the *xy* dimension with a 1.8 μm interval between optical sections in the *z*-dimension. Confocal *z*-stack images of the dorsal retinal margin of juvenile zebrafish were captured with a Leica TCS SP8 LSCM (Leica Microsystems, Wetzlar, Germany) and Leica 40X PL APO CS2 Water Immersion lens. Individual GFP-labeled Müller glia (1 to 5 per experimental sample) were targeted in each retina with 700–900 nm diameter circles created by the ‘Region of Interest’ tool centered on the radial processes at the level of a single optical section 25–50 μm below the OLM. Ablation was performed with the Chameleon 2-photon Ti:Sapphire laser tuned to 800 nm at maximum power output with acquisition speed of 10 Hz and zoom factor 12×.

Confocal *z*-stack images of the Müller glial GFP reporter were selectively projected using the MATLAB routine described above under Cell Counts. The program reconstructs the OLM surface three-dimensionally by averaging the fluorescent signal with a Gaussian kernel in the *z*-direction and interpolating in the *x-* and *y-*directions, and selectively capturing the fluorescent signal only at the OLM. The reconstructed surface was also used to estimate and correct for inclination of the OLM plane due to sample tilt. Radial fibers of Müller glial cells were masked manually when needed to achieve a cleaner projection. The centroid positions **r**_*i*_ = (*x*_*i*_, *y*_*i*_)^*T*^ of photoreceptor profiles surrounding the lesion area were then tracked in ImageJ and imported to MATLAB. Sample tilt was corrected by estimating the orientation of the OLM from the projection algorithm and rotating **r**_*i*_. Tissue deformation was estimated by finding the affine transformation ${\widehat{\mathbf{r}}}_i^f={\mathbf{Mr}}_i^0+\kern0.5em \mathbf{b}$ of the initial photoreceptor positions ${\mathbf{r}}_i^0$ that minimizes the mean-squared deviation $\sum \limits_i{\left\Vert {\mathbf{r}}_i^f-{\mathbf{Mr}}_i^0-\kern0.5em \mathbf{b}\right\Vert}^2$ between the transformed positions ${\widehat{\mathbf{r}}}_i^f$ and the actual positions ${\mathbf{r}}_i^f$ of the same photoreceptors at a given time after ablation. The residuals after this transformation at 0–15 min, 15–30 min, and >30 min after ablation are approximately 13%, 19%, and 23% of typical neighboring cell distance, respectively, indicating that the predominant deformation is indeed affine. For control experiments, the first two corresponding figures are 7% and 11% (there is no control with >30 min after ablation). Rotation of the images across samples was corrected by computing the polar decomposition **M** = **UP**, where **U** is unitary and **P** is positive semi-definite. Strain magnitude and orientation were then calculated as the eigenvalues and eigenvectors of **P**, with the eigenvector approximately along the cone columns identified with the *y-* strain and the eigenvector approximately perpendicular to the columns as the *x*-strain. For statistical analysis, strains at all different times after ablation were grouped together and compared against control.

## Results

### Remodeling of apical profiles at the retinal margin reflects dynamic and gradual maturation of the lattice mosaic

Apical epithelial profiles of photoreceptors (rods and cones) and Müller glial processes at the level of the outer limiting membrane (OLM) [28, 29] can be visualized by immunostaining for the zonula adherens scaffolding protein, Zonula Occludens 1 (ZO1) in retinal flat-mounts from adult zebrafish [10] (Fig. 1A and B). (The identity of these apical profiles has been verified previously in transgenic zebrafish lines with fluorescent reporters driven by promoters specific for Müller glia (*gfap*), rods (*rhodopsin*), cones (*cone alpha transducin*), UV cones (*sws1 opsin*)*,* Blue cones (*sws2 opsin*), and Red cones (*trβ2*) [10, 14, 25].) The profiles of each cell type have distinctive shapes and positions, and thin lamellar processes of Müller glia completely enwrap each photoreceptor at the level of the OLM [29, 31].

Fig. 1C illustrates the alignment of the photoreceptor lattice mosaic on the hemispheric retinal surface of a larval zebrafish (3-weeks-old, 0.72 cm standard length, exclusive of the tail) at a stage soon after photoreceptors begin to organize into a lattice mosaic. Rows of Blue cones, labeled with a fluorescent reporter (*sws2:*mCherry), align orthogonal to the retinal margin. (With only Blue cones labeled, rows are much more apparent than columns because the distance between adjacent Blue cones is less along a row than within a column, see Fig. 1B.) At the retinal margin cohorts of immature, differentiating cones are incorporated into the pre-existing mosaic appositionally as a new column, which can be visualized by standard cell birth-dating methods. One-month-old juvenile zebrafish were treated systemically for 4 h with EdU to label mitotic retinal progenitor cells, and the newly generated, EdU-labeled photoreceptors were allowed to differentiate. After 10 days, retinal flat-mounts were processed for EdU detection, which revealed a ring of EdU-labeled cones (and other retinal neurons) approximately 80 μm from the margin (Fig. 1D). Counting the number of cone columns between the EdU ring and the retinal edge revealed that 2.5 to 3.5 single cell width columns of cones were added to the lattice mosaic each day, with higher growth rates dorsally than ventrally (Fig. 1E). Retinal growth continues in juvenile zebrafish, slowing down in adults after ~ 4 months (Fig. 1F); we focused our analysis on 1–2-month-old juveniles, whose retinas were growing rapidly.

The precise spatiotemporal pattern of retinal growth allows us to examine in a single preparation successive stages in building the multiplex photoreceptor lattice mosaic. To systematically describe how the photoreceptor lattice mosaic is established from proliferative retinal progenitors at the retinal margin, we imaged retinal flat-mounts with ZO1 immunocytochemistry to visualize the apical epithelial profiles of photoreceptors and Müller glia. Cell profiles at the retinal margin show gradual and dynamic changes from proliferative, to differentiating, to patterned zones (Fig. 1G, Additional file 2: Figure S1 A and Additional file 3: Movie S1). To minimize surgical damage to the retina, this preparation retains the overlying, but closely apposed retinal pigment epithelium (RPE) (Fig. 1G”’, dashed polygon; Additional file 2: Figure S1 B). Retinal progenitors in the proliferative germinal zone, identified with a mitotic cell marker, pH 3 (Fig. 1G”’ and Additional file 2: Figure S1 B’ and 1 B”), are heterogeneous in size and shape with flattened boundaries (Fig. 1G and G”’). These dividing cells represent both multipotent and committed retinal progenitors that together generate all types of retinal neurons and Müller glia [12, 26, 45]. At the central border of the pH 3-labeled zone, apical profiles become restricted to photoreceptors and glia, as committed inner retinal neurons detach and migrate basally [25, 46]. The transition between proliferating and differentiating zones is represented by reduced sizes and rounded shapes of post-mitotic, developing photoreceptors (Fig. 1G and G”, white dots,) and irregular, sharply polygonal shapes of Müller glia (Fig. 1G and G”, asterisks). An ordered lattice mosaic is not yet apparent and immature cones have not yet begun to express specific opsin genes, which occurs relatively late in the gradual process of photoreceptor differentiation [25, 47]. In the adjacent, more central zone, the lattice mosaic pattern emerges (Fig. 1G and G’).

### Precise spatial arrangement and cone cell fate commitment precedes column organization

**Fig. 2:**
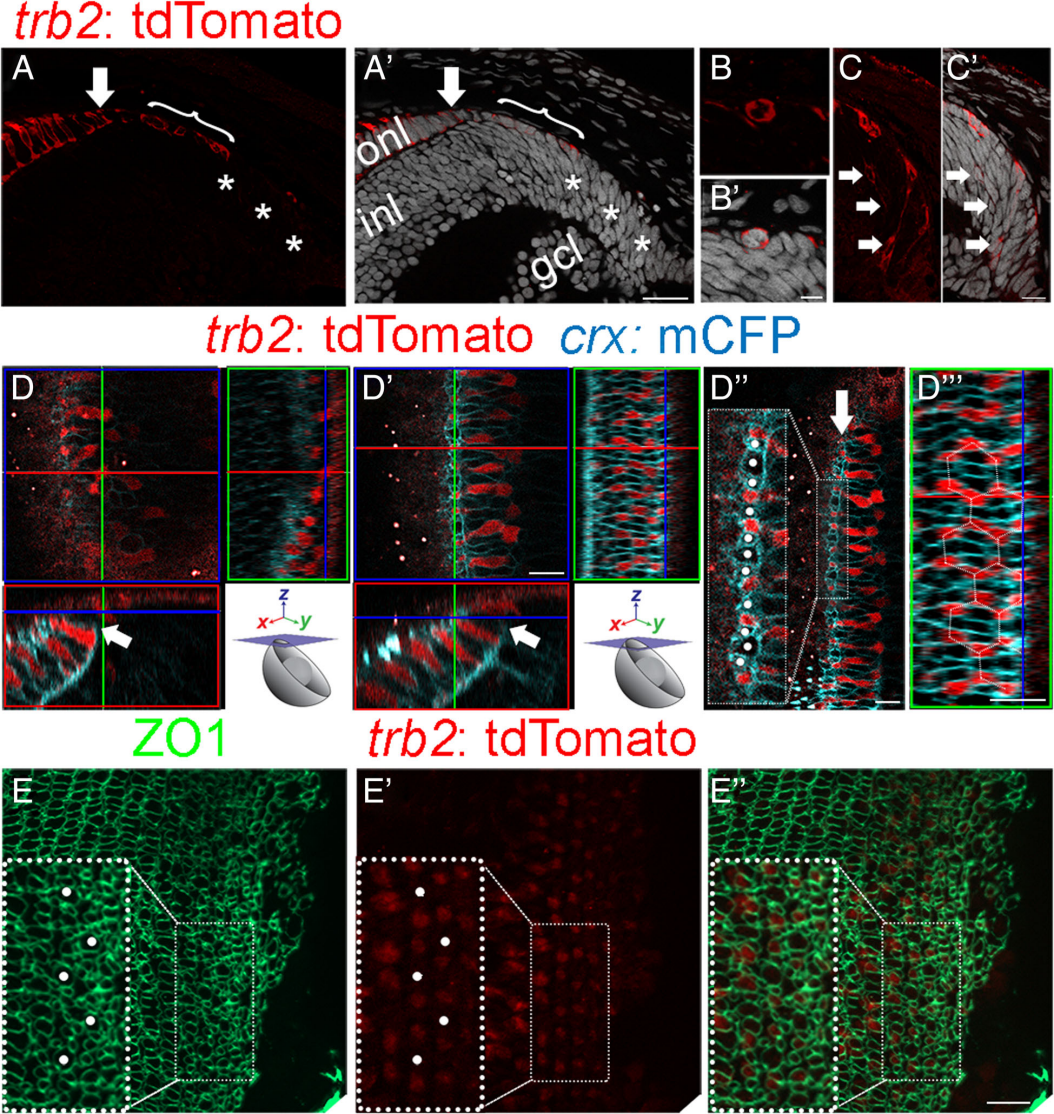
Spatial arrangement of cones is already patterned in the differentiating pre-column zone. (A-C) Retinal cross-section, juvenile zebrafish, *Tg*(*trβ2*: tdTomato) (red). Nuclei stained with Hoechst (gray). (A, A’) Proliferating retinal progenitors in the germinal zone (asterisks) and differentiated retina to the left: ganglion cell layer (gcl), inner nuclear layer (inl), and outer nuclear layer (onl). Immature, post-mitotic tdTomato+ Red cones (arrow) in the photoreceptor layer (onl) adjacent to Red cone progenitors (bracket) at the apical surface. (B, B’) Mitotic tdTomato+ progenitor (grey). (C, C’) Most peripheral tdTomato+ cells migrate basally toward inner retina (arrows). (D-D”’) Live-imaging 3D reconstruction of dorsal retinal margin of a *trβ2*: tdTomato (red);*crx:* mCFP (cyan);*ruby* juvenile fish. (See Additional file 4: Movie S2 for complete *z-*stack series.) (D) Single *z*-level focal plane (blue box); *xy-*crosshair is on tdTomato+ cell located in the proliferative germinal zone. (See also Additional file 4: Movie S2, white asterisks.) The flanking panels are slice views of the 3D reconstruction in the *yz* plane (red box), perpendicular to the retinal margin and the *xz* plane (green box), planar view of the retinal surface. (D’) Single, deeper *z*-level focal plane; *xy-*crosshair on a tdTomato+ immature Red cone in first cone column. In the *yz* plane (red box), axonal processes of immature cones separate the onl and inl (cyan, arrow). In the *xz* plane (green box), the *xz* -crosshair is on a Red cone in the first column. (D”) Higher magnification view in the *xy* plane of the first cone column (arrow); rounded, immature cone profiles (white dots). (See also Additional file 4: Movie S2.) (D”’) Higher magnification view in the x*z* plane of Red cones in first column (*xz*-crosshair) incorporated into the hexagonal pattern of the mature mosaic. (E-E”) Retinal flat-mount of a *Tg*(*trβ2*: tdTomato) fish (red) with anti-ZO1 (green). (E) Maximum intensity *z-*projection of ZO1 (green). (E’) Single *z-* focal plane at ZO1 level of *trβ2:*tdTomato label in the pre-column zone. Large immature UV cone profiles (white dots in the inset) centered in Red cone hexagons. (E”) Merged image. Scale bars: 20 μm (A’); 5 μm (B’); 10 μm (C’, D”, D”’, E”)

To understand how cell packing, spatial arrangement, and cell identity are coordinated as new cones are incorporated into the lattice mosaic, we used transgenic reporter lines. In zebrafish embryos, the *thyroid receptor beta 2* (*trβ2*) promoter drives expression in a subset of retinal progenitors that give rise exclusively to selected types of inner retinal neurons and to Red cones, but transgene expression is retained after differentiation only in Red cones [25]. We first asked whether expression of this reporter is similar in juvenile *Tg(trβ2*:tdTomato*)* zebrafish. As expected, only a subset of proliferative retinal progenitors are *trβ2*:tdTomato+ (Fig. 2A – C), including mitotic figures (Fig. 2B and B’), visualized in radial histological sections. In the germinal zone (Fig. 2A and A’, asterisks), *trβ2*:tdTomato+ retinal progenitors are in regions where retinal lamination has commenced, and inner retinal neurons are differentiating (Fig. 2C and C’ arrows), consistent with the embryonic lineage of *trβ2*:tdTomato+ progenitors, which includes horizontal cells and ganglion cells [25]. The more centrally located *trβ2*:tdTomato+ cells with cuboidal nuclei at the apical surface are within the nascent photoreceptor layer in the differentiating zone (Fig. 2A and A’ bracket) and are developing Red cones [47]. Other retinal neurons in the *trβ2*:tdTomato lineage migrate to more basal retinal layers and lose the *trβ2*:tdTomato label (Fig. 2A, A’) [25].

To provide a more accurate, in situ view of the immature Red cones, we developed a live imaging technique with multi-photon confocal microscopy and generated 3D reconstructions of the dorsal retinal margin in young juvenile fish. For this analysis, we crossed *Tg(trβ2*: tdTomato) and *Tg(crx:*mCFP), a membrane-targeted label expressed by all photoreceptors [25, 48], into the semi-transparent ‘ruby’ mutant background [40]. With this method we can image living anesthetized fish only up to ~ 1 h without compromising viability. (Compared to embryonic zebrafish, which can be imaged for longer period of hours or days [24–26], our juvenile animals have a larger body size and scales that reduce gas exchange over the integument, both of which limit the viability of immobilized fish.) Unfortunately, extended time-lapse studies to trace the cell lineage of the *trβ2*:tdTomato+ cells and the dynamics of lattice mosaic formation are thus not presently feasible. Nevertheless, the precise spatiotemporal pattern of retinal growth allows all stages in formation of the lattice mosaic to be visualized. The images in Fig. 2D are 3D–reconstructions of a confocal z-stack (see insets in Fig. 2D and D’). In the proliferative zone, *trβ2*:tdTomato+ progenitors (e.g.*,* Fig. 2D, cell indicated by the red-green cross-hair) are not regularly spaced (also see Additional file 4: Movie S2, asterisks) and are peripheral to the onset of retinal lamination and formation of the outer nuclear layer of photoreceptors (Fig. 2D, red box, white arrow; also see Fig. 2 C, C’). A regular spatial arrangement of *trβ2*:tdTomato+ cells first appears in the pre-column zone (e.g.*,* Fig. 2D’, cell indicated by the red-green cross-hair) and only in cells at the apical surface in the nascent photoreceptor layer (Fig. 2D’ and D”; also see Fig. 2A, A’, bracket). Note that nearest neighbor Red cones in the mature lattice mosaic form a hexagonal pattern (Fig. 1B), and this hexagonal pattern of *trβ2*:tdTomato+ cells is apparent in the planar (surface) retinal view in the 3D reconstruction (Fig. 2D’ green box, shown at higher magnification in D”). Slicing through the 3D–reconstruction in the radial dimension orthogonal to the retinal margin (Fig. 2D’, red box) reveals that these *trβ2*:tdTomato+ cells are immature Red cones with axons beginning to form the outer plexiform layer, which is defined by a dense band of *crx*:mCFP labeling basal to the nuclei (Fig. 2D’ arrow). Finally, slicing through the 3D–reconstruction in the radial dimension parallel to the retinal margin, as indicated by the green box in Fig. 2D’ and shown at higher magnification in D”’, shows that these immature Red cones are incorporated into the newest formed, single cell width cone column. These immature cones have not yet developed an apical inner and outer segment, structures prominently labeled by mCFP in more central, differentiated cones (Fig. 2D’, red box lower left quadrant; see also Additional file 4: Movie S2).

In the first column of immature cones to emerge at the margin, successive *trβ2*:tdTomato+ Red cones are separated alternately by one or three cell profiles (Fig. 2D”’ and Additional file 4: Movie S2). This pattern is consistent with the mature lattice mosaic, in which adjacent Red cones in a column are separated, alternately, by one Blue cone or a set of three cones, Green-UV-Green (Fig. 1B). Although specific markers for progenitors of zebrafish cone subtypes other than Red cones have yet to be identified, the regularity of this pattern supports the suggestion that all of these immature cones may have already correctly committed to a specific identity. Additionally, the central profile in the triplets is typically larger (Fig. 2D” and Additional file 4: Movie S2), which is a characteristic of UV cones in the dorsal retina [10, 14]. The spatial arrangements of *trβ2*:tdTomato+ Red cones and *crx:*mCFP+ cone profiles in the newest cone column are also correctly aligned with the adjacent column (Fig. 2D”’ and Additional file 4: Movie S2), according to the organizational ‘rules’ of the lattice mosaic (Fig. 1B), and immature Red cones are already incorporated into the hexagonal pattern of Red cones in the lattice mosaic (Figs. 1B and 2D”’). The hexagonal pattern of immature Red cones in the pre-column zone is also apparent in flat-mount *trβ2*:tdTomato retinas labeled with ZO1 (Fig. 2E-E”). Note again the single large profiles of UV cones located in the middle of each Red cone hexagon (Fig. 2E-E”, white dots), whose identity is confirmed with the UV reporter, *sws1*:EGFP (Additional file 5: Figure S2 A and A’). These results together indicate that the correct spatial arrangement of Red and UV cones (and potentially, Blue and Green cones) is present when, or soon after, they acquire their subtype identities and prior to packing into a regular lattice mosaic.

### Planar-polarized Crumbs distribution appears just prior to columnar packing

**Fig. 3:**
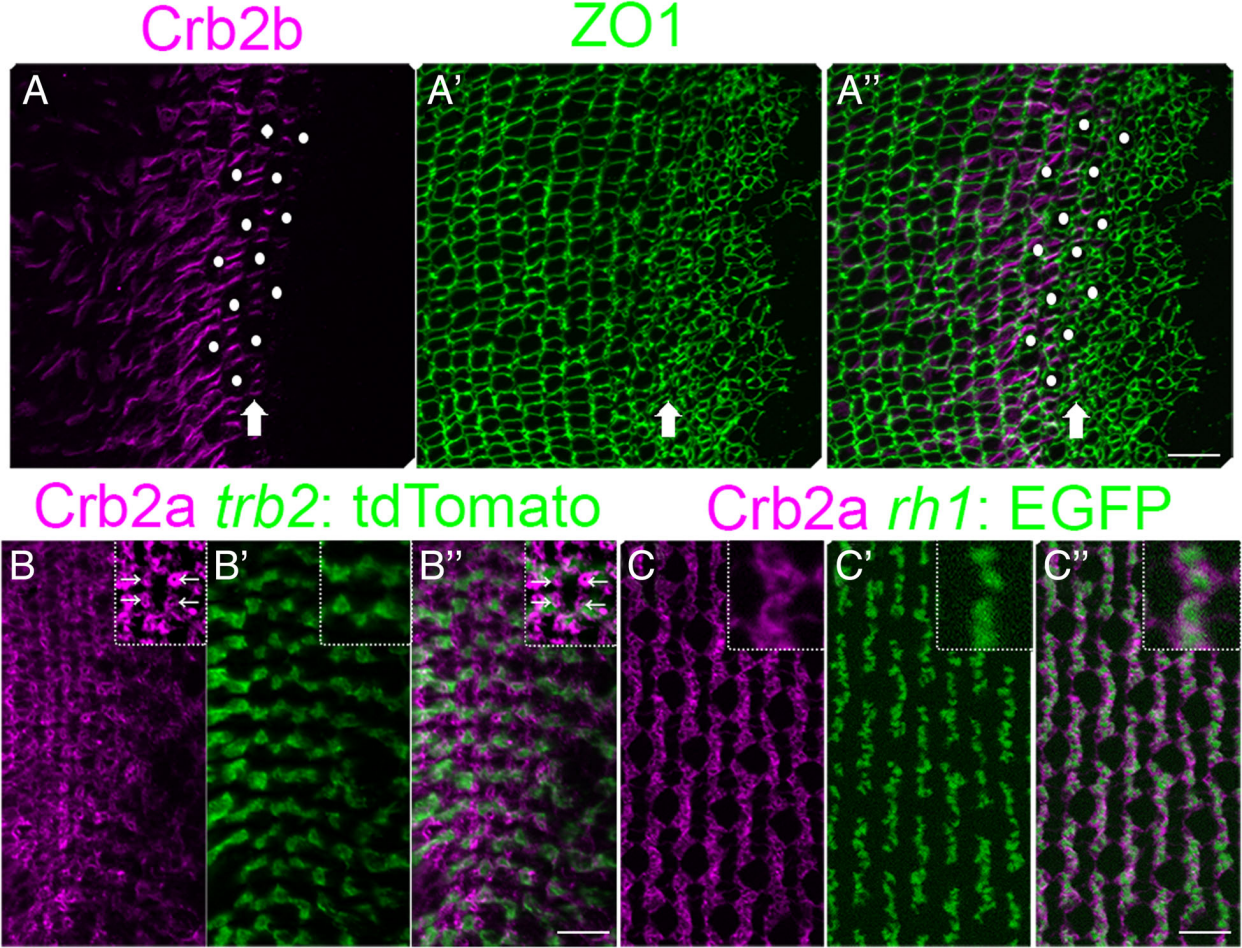
Spatial patterning of cones precedes planar polarized Crumbs localization. (A-A”) Retinal margin in a flat-mount preparation, immunolabeled for Crb2b (magenta) and ZO1 (green). (A) Single *z-*level focal plane of the Crb2b channel at the level of ZO1 staining (OLM) in the pre-column zone. Crb2b ‘ladders’ (arrow) in the pre-column zone and adjacent columns. (Note that due to curvature of the retinal surface, the equivalent ZO1 level in the central retina at the left is at a deeper focal plane.) Immature UV cone profiles (white dots) in the pre-column zone. (A’) The maximum intensity *z-*projection of ZO1 labeling. (A”) Merged image. (B-B”) Retinal margin in a flat-mount preparation, immunolabeled for Crb2a (magenta) with the *trβ2*: tdTomato (green) Red cone marker. (B) Note small rings of strong Crb2a staining between cone columns (inset, arrows). (B’, B”) The intensely-labeled Crb2a+ rings are not co-labeled with *trβ2*: tdTomato. (C-C”) Immunoreactivity for Crb2a (magenta) with the *rh1*: EGFP (green) rod marker. (C’, C”) The strongly immunoreactive Crb2a+ rings surround *rh1:* EGFP+ labeled rod photoreceptors. Scale bars: 10 μm (A”, B”, C”)

In the mature lattice mosaic, Crb2b proteins show planar polarized localization and homophilic adhesion at intra-column, but not inter-column, interfaces of the apical inner segments of Red, Green, and Blue cone photoreceptors [10, 27]. Crb2b is not expressed in UV cones, rods, or Müller glia, although Crb2a is expressed in all these cell types [27]. In planar views, distribution of Crb2b immunoreactivity, which is localized to the subapical membrane of the cone myoid, creates ‘ladders’ of four parallel segments separating pentameric units of Green-Red-Blue-Red-Green cones [10, 27]. In the growing retinal margin, Crb2b ‘ladders’ appear nearly simultaneously in immature cones immediately adjacent to the first contiguous column (Fig. 3A-A”, arrow). This restricted, planar polarized Crb2b at cone-cone interfaces indicates that immature Red, Green, and Blue cones have acquired their identity and correct planar directionality prior to columnar packing. However, polarized Crb2b adhesive interactions occur after the initial differentiation and spatial patterning of Red and UV cones, as shown by regularly-spaced, large UV profiles located peripheral to the Crb2b ladders (Fig. 3A-A”, white dots). These observations suggest that the initial spatial patterning of cones in the pre-column zone involves a Crb2b-independent mechanism.

### Immature rod photoreceptors are correctly positioned in the pre-column zone

Interestingly, planar polarized Crb2a ‘ladders’ are not seen in the differentiating, pre-column zone, even though planar polarized distribution of both Crb2b and Crb2a in apical processes of Red, Green, and Blue cones is a prominent feature of the mature lattice mosaic [10]. Instead, the most prominent Crb2a immunostaining in the pre-column zone comprises four small, circular profiles inside the *trβ2*:tdTomato+ Red cone hexagons surrounding a central UV cone (Fig. 3B-B”, inset arrows). This spatial location is consistent with the initial position of rods in the larval zebrafish retina [49]. The Crb complex mediates rod photoreceptor apical morphogenesis in developing zebrafish retina [50], and in mature retina, strong Crb2a immunoreactivity at the OLM surrounds profiles of rods labeled with the rhodopsin transgene, *rh1:* EGFP (Fig. 3 C-C”). Although onset of transgene expression driven by the rhodopsin promoter occurs later in rod differentiation, together these data strongly suggest that immature rod photoreceptors are also precisely positioned prior to packing into a regular lattice mosaic.

To further evaluate the presence of rods in the differentiating pre-column zone, we analyzed the ratio of *trβ2*:tdTomato+ (Red cones) to *trβ2*:tdTomato*-* (other cone types and rods) visualized by their ZO1 profiles in retinal flat-mounts. We predict a 1:2 ratio of *trβ2*:tdTomato+ to *trβ2*:tdTomato- profiles, if only cones, but not rods, are present (Additional file 6: Figure S3 A and B). However, if four rods are present at the corners of each UV cone, then the predicted ratio of *trβ2*:tdTomato+ to *trβ2*:tdTomato- profiles is 1:4 (Additional file 6: Figure S3 A and B). With an automated counting method cell profiles were segmented and classified as *trβ2*:tdTomato+ or - (Additional file 7: Figure S4 A-A”’). The measured ratio of *trβ2*:tdTomato+ to *trβ2*:tdTomato- profiles was 1: 4.87, *n* = 2 retinas, 780 total cells (Additional file 7: Figure S4 C); samples from three additional retinas were counted by visual inspection, and ratios were within the range of the automated samples. These results support the inference that rods are present in the pre-column zone. These results imply correct spatial coordination of rods and cones, even though a lattice mosaic is not yet present.

### Spatial analysis of Müller glial processes reveals parallel bands prior to formation of cone columns

**Fig. 4:**
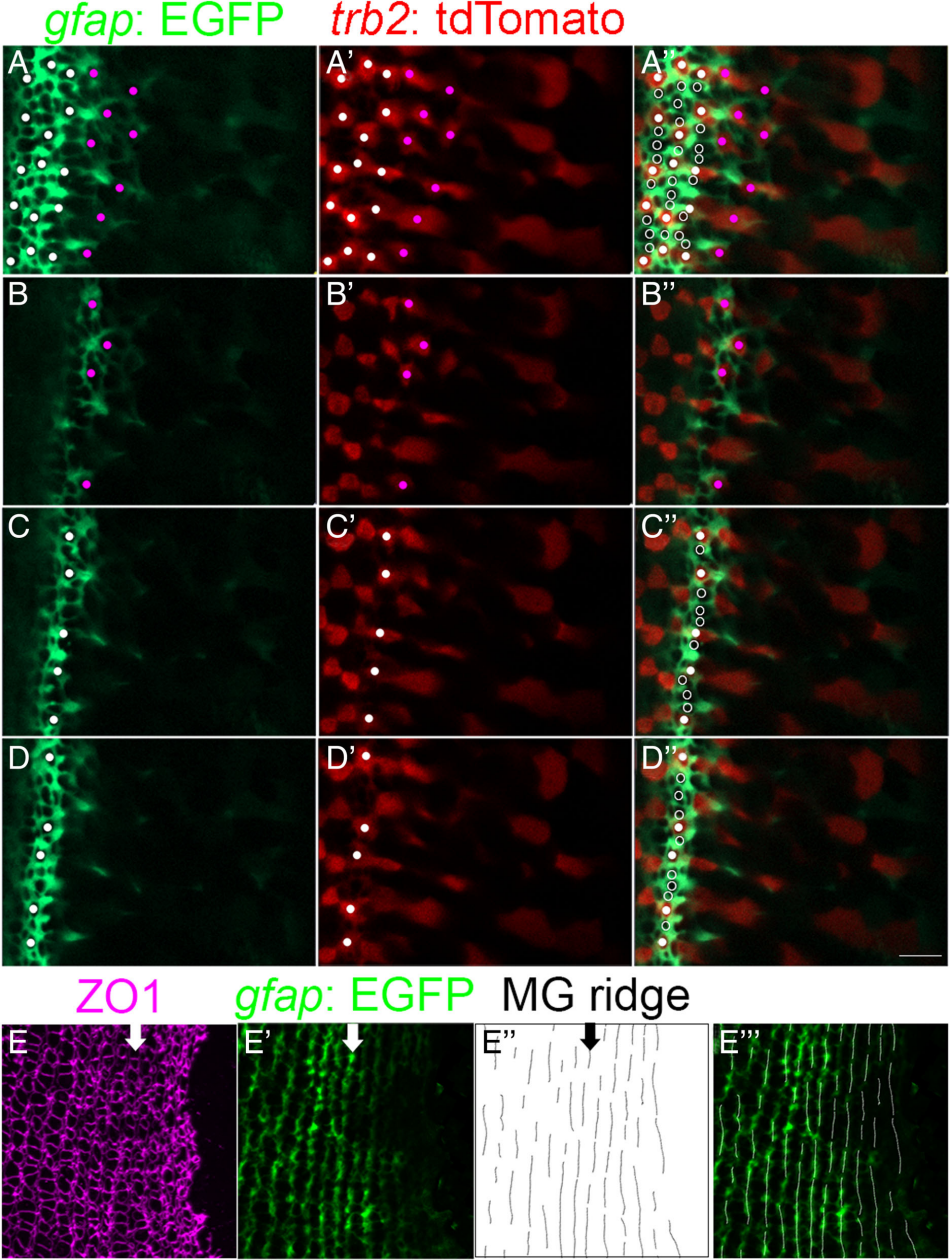
Parallel bands of Müller glial scaffolding appear simultaneously with differentiating cones. (A-D) Live imaging of the retinal margin, with Red cone *trβ2*:tdTomato (red) and Müller glial *gfap:*EGFP (green) markers. Glial processes completely surround cone and rod photoreceptors, which can be visualized as round ‘holes’ in the GFP label at the level of the OLM. (A-A”) Maximum intensity *z-*projection. Red cones in the patterned lattice mosaic (white dots) and pre-column zone (magenta dots). (A”) Red cones (white dots) and non-red cones (open white dots) in the patterned lattice mosaic. (Also see Additional file 9: Movies S3 and Additional file 10: Movie S4.) (B-D) Single *z*-levels focused on the pre-column (B), and patterned (C, D) areas. (E-E”’) Ridge analysis applied to Müller glial profiles in flat-mount of *Tg*(*gfap*: EGFP) (green) retina immunolabeled for ZO1 (magenta). (Also see Additional file 7: Figure S4.) Arrows (E-E”) indicate the location of the first ordered column of cone photoreceptors that can be clearly discerned from cell outlines at the level of the OLM (E’). Parallel ridges of Müller glia extend for several iterations into the pre-column zone (E’-E”’). Scale bar: 5 μm (D”)

Müller glial apical processes at the OLM expand to form both homotypic adherens junctions between glial processes and heterotypic adherens junctions with photoreceptors [10, 29]. In retinal flat-mounts, glial processes, identified with the *gfap:* EGFP reporter, surround rod and cone profiles, forming thin intra-column lamellae, and thicker inter-column expansions (Additional file 8: Figure S5). To examine the temporal coordination between morphological maturation of glial processes and emergence of the hexagonal pattern of Red cones, we imaged living, juvenile, *Tg*(*gfap:* EGFP*; trβ2:* tdTomato;*ruby*) fish. Immature glia express GFP weakly in the peripheral proliferative zone, and GFP signal increases as glia differentiate and expand their apical processes laterally at the OLM (Fig. 4A-D and Additional file 9: Movies S3 and Additional file 10: Movie S4). The repeating pattern of one and three immature cones between successive Red cones, seen with the *crx:*mCFP reporter (Fig. 2D), can also be visualized by glial processes that surround the immature cones (Fig. 4 A”, C”, D”, white filled and open dots). These observations suggest that apical Müller glial processes provide a scaffold around differentiating photoreceptors at the level of OLM in the pre-column zone.

In the mature lattice mosaic, glial processes preferentially distribute into parallel, inter-column bands (Additional file 8: Figure S5), so we asked whether a similar distribution exists in the pre-column zone. With ZO1 immunoreactivity (Fig. 4E) to selectively capture the GFP-labeled glial profiles at the level of the OLM (Fig. 4E’), we applied a ridge analysis algorithm (Fig. 4E”) [44] that uses steerable filters to detect the presence and orientation of glial bands. Tuning the width of the filter revealed variations in the thickness of glial bands. We found that not only are glial bands that occupy spaces between adjacent columns consistently thicker, they also have a consistent direction parallel to the mature columns, even in the pre-column region where differentiating photoreceptors are not yet aligned (Fig. 4E” and E”’). These results suggest that a glial scaffold contributes to spatial alignment of photoreceptors into parallel columns.

### Differentiating cones lose N-cadherin expression, whereas N-cadherin adhesive interactions between Müller glia are enhanced in the mature lattice mosaic

If glial processes participate in organizing the spatial arrangement of cones in the pre-column zone then heterotypic intercellular interactions are required. N-Cadherin transcripts (*cdh2*) and protein are expressed by retinal progenitors in the germinal zone and by differentiated glia, but not by mature photoreceptors [45, 51]. Retinal progenitors form zonula adherens junctions through homophilic intercellular N-cadherin-mediated adhesion [29]. We predicted that immediately after withdrawing from the cell cycle, post-mitotic, immature cones transiently retain N-cadherin expression, so that during their initial phase of differentiation, they participate in N-cadherin-mediated intercellular junctions with glia.

**Fig. 5:**
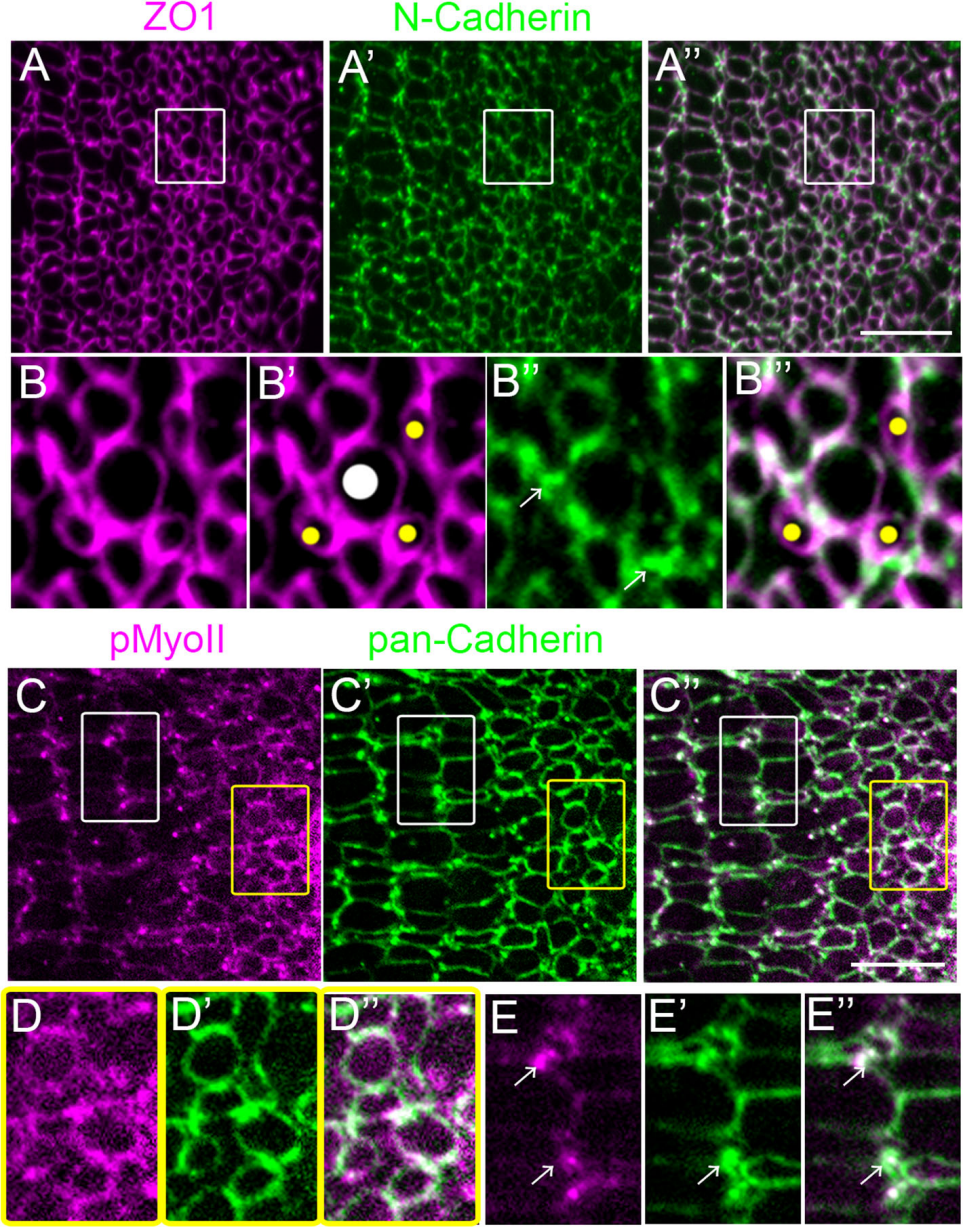
N-cadherin is associated with actomyosin mechanical forces in differentiating cones in the pre-column zone and with Müller glia inter-column bands in the mature mosaic. (A-A”) Flat-mount of retinal margin, immunolabeled for ZO1 (magenta) and N-Cadherin (green). (B-B”’) Higher magnification of pre-column zone (boxes in A-A”). N-Cadherin labeling surrounds round cone profiles and is concentrated in adherens junction puncta (arrows) outside photoreceptor profiles. Immature rods are outlined by ZO-1 (B’, B”, yellow dots) but are negative for N-cadherin (B”). Large round profiles represent immature UV cones (B’, white dot). (C-C”) Flat-mount of retinal margin, immunolabeled for phospho-Myosin light chain II (magenta) and pan-Cadherin (green). (D-D”) Higher magnification of pre-column zone (yellow box); strong pMyoII and pan-Cadherin staining of round profiles of differentiating cones. (E-E”) Higher magnification of mature lattice mosaic (white box); pMyoII and pan-Cadherin co-localize to puncta between cone columns (arrows). Scale bar: 10 μm (A” and C”)

We therefore examined expression and distribution of N-Cadherin during maturation of the lattice mosaic. With in situ hybridization on retinal cross-sections we detected *cdh2* transcripts in the germinal zone and in adjacent immature cones (Additional file 11: Figure S6 A-A”, arrow), but as expected, not in differentiated photoreceptors (Additional file 11: Figure S6 A, A’, A”’). In the mature retina, *cdh2* transcripts were restricted to Müller glia (Additional file 11: Figure S6 A-A”, arrowheads). Consistent with *cdh2* expression, in retinal flat-mounts, N-Cadherin immunoreactivity was detected at the level of the OLM in the rounded profiles of differentiating cones in the pre-column zone (Fig. 5 A-A”, B-B”). Strong punctate staining of ZO1 co-localized with N-cadherin was apparent in inter-column bands in the mature mosaic (Fig. 5 A-A”, two cone columns are visible at the far left side of the image) and in the pre-column area (Fig. 5 B” arrows), consistent with expression of N-Cadherin in homophilic adherens junctions between glia. In the pre-column zone, a subset of ZO1-labeled apical profiles was negative for N-Cadherin (Fig. 5 B’ and B”’, yellow dots). These small, round N-Cadherin-negative ZO1 profiles are likely to be immature rod photoreceptors, based on their location at each corner of the large UV cones. The lack of N-Cadherin expression in immature rods is consistent with their production from committed rod progenitors, which lack apical processes and adherens junctions and which divide mitotically in the outer nuclear layer [52, 53].

Intracellularly, N-Cadherin links to actomyosin via catenin complexes to regulate cytoskeletal contractility that controls cell shape and position, mediated by phosphorylation of Myosin light chain II [54, 55]. We therefore examined immunolocalization of activated phospho-MyosinII (pMyoII). In the pre-column zone, pMyoII co-labeled with Cadherin in rounded profiles of immature cones (Fig. 5C-C” and D-D”, yellow box). In the mature lattice mosaic, pMyoII predominately co-localized with Cadherin puncta in glial processes between cone columns (Fig. 5C-C” and E-E”, white box, arrows). These results suggest that immature cones and glia in the pre-column zone remodel their apical profiles through Cadherin-catenin-mediated actomyosin contractility, and that in the mature lattice mosaic, Müller glial processes generate contractile forces in parallel bands between cone columns.

### Anisotropic tension in Müller glia is aligned with the photoreceptor lattice mosaic

Previous work predicted that highly anisotropic mechanical stresses within the retinal epithelium mediate formation and maintenance of the lattice mosaic [10]. These studies further pointed to planar polarized adhesive interactions mediated by Crb2a and Crb2b between adjacent cones along a column as one possible source of this anisotropy [10, 27]. However, delayed onset of Crumb2a/2b ‘ladders’ in the retinal margin indicates that additional factors are involved in lattice mosaic formation. The distribution of activated pMyoII in puncta representing N-Cadherin-mediated adherens junctions in inter-column bands of glia suggests that actomyosin-mediated contractile forces in Müller glia might also be anisotropic.

**Fig. 6:**
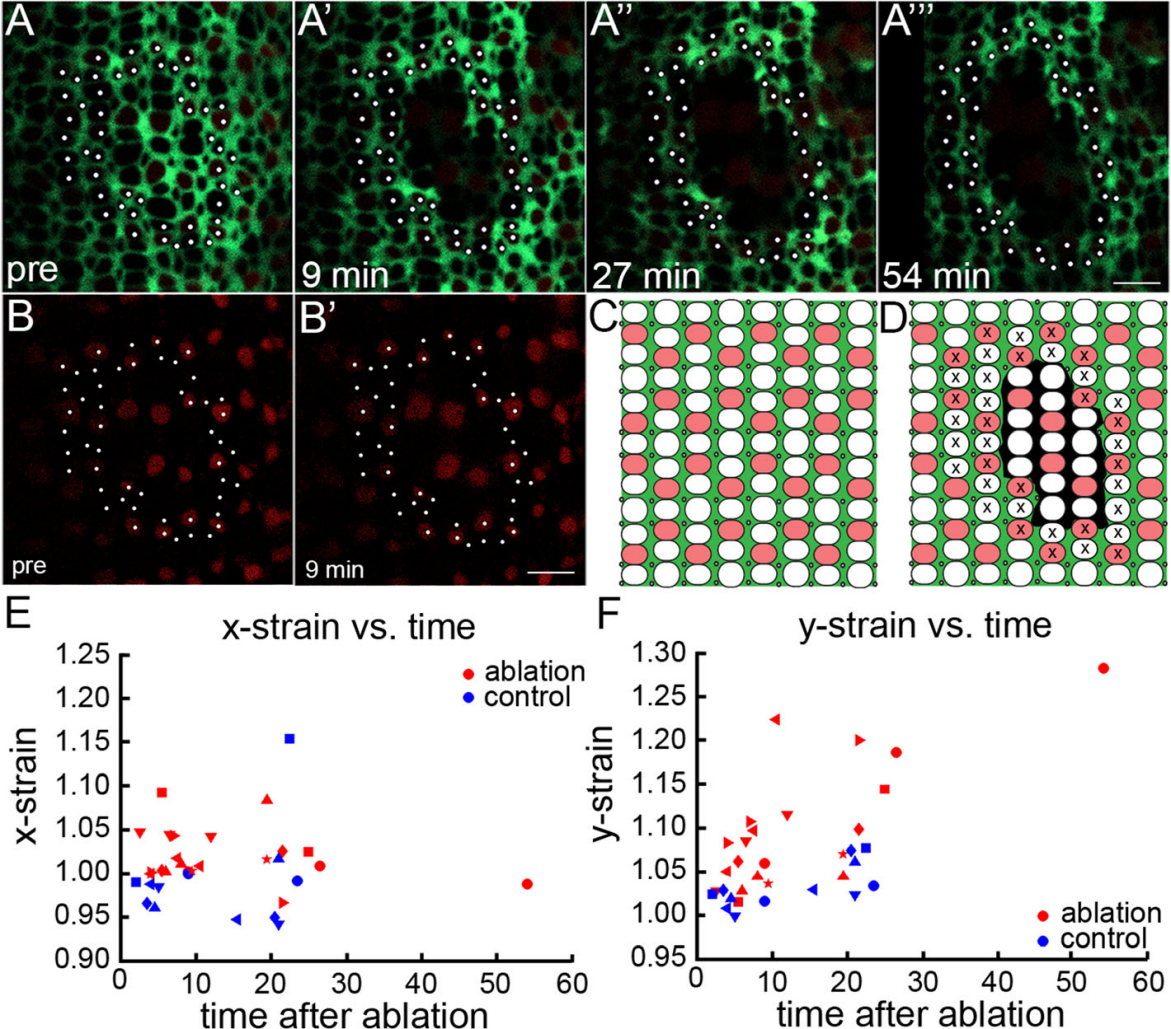
Müller glia mediate anisotropic mechanical forces in the retinal epithelium. (A-A”) Live imaging time course of targeted Müller glial ablations in *Tg*(*gfap*: EGFP; *trβ2*: tdTomato;*ruby*) juvenile zebrafish with glial (green) and Red cone (red) reporters. Photoreceptor profiles tracked for strain analysis (white dots). (A’-A”’) Targeted ablation of Müller glia introduces a hole in the sheet of Müller glial processes at the OLM. (Also see Additional file 12: Figure S7.) Relaxation of surrounding retinal epithelium at 9 min (A’), 27 min (A”), and 54 min (A”’) after ablation. (Also see Additional file 13: Movie S5.) (B-B’) *trβ2*: tdTomato+ Red cones survive after Müller glial ablation. (C-D) Schematic of pre (C) and post (D) ablation. Cones tracked for strain analysis (x). (E-F) Mechanical strain perpendicular (*x-*strain, E) and parallel (*y*-strain, F) to the retinal margin after ablation. Strains greater and smaller than 1 represent stretching and compression, respectively. Each retina is represented by a different shaped symbol (*n* = 6 controls; *n* = 8 experimental). Horizontal axis is the time interval between the ablation and the middle of the post-ablation imaging scan; two or three post-ablation scans were collected for each retina. Scale bars: 5 μm (A”’ and B’)

To test this hypothesis, we applied multi-photon laser ablation to destroy Müller glia in living juvenile *ruby* transgenic zebrafish carrying the *gfap:*EGFP reporter. We used the *trβ2*: tdTomato reporter to monitor maintenance of Red cone photoreceptors and to ensure that the ablation was restricted only to Müller glia. We targeted one or more individual radial Müller glial processes at a *z-*level near the cell body in the inner nuclear layer, and we adjusted the laser power to destroy the entire cell and selectively abolish the apical glial processes (Additional file 12: Figure S7). We observed no return of fluorescent signal with repeated imaging over intervals up to ~ 1 h, suggesting that the loss of GFP signal was not due to photobleaching. The targeted cell ablation created a hole in the sheet of glial processes at the level of the OLM (Fig. 6A-A’ and C-D and Additional file 12: Figure S7) without damaging photoreceptors. Red cones, which had lost direct contacts with glia, nonetheless maintained their integrity and relative positions within the hexagonal pattern (Fig. 6B and B’). We then examined relaxation of the retinal epithelium after glial ablation by tracking positions of photoreceptors immediately surrounding the hole over a period of several minutes (Fig. 6A-A”’). Importantly, tracked photoreceptors were completely surrounded by intact glial processes, implying that their motion reflected a global mechanical relaxation, not a specific response to loss of glial contacts. As a control, we repeated the same procedure, but with the laser turned off at the ablation stage.

We examined 8 ablated and 6 control retinas and measured the tissue deformation (strain) perpendicular (*x*-strain) and parallel (*y*-strain) to the retinal margin (Fig. 6E and F, respectively). After glial ablation, we found no significant difference in strain perpendicular to the margin around the ablation site (Welch’s unequal-variance, *p*-value = 0.1128). Parallel to the margin, however, measures of *y-*strain indicated significantly more elongation even at the earliest time-point (~5 min after ablation), and the amount of elongation increased with time (*p*-value = 0.0008). Confirming this, Hotelling’s multivariate unequal-variance test using both *x*- and *y*-strain shows significant (*p* = 0.0002) difference between ablation and control groups. Anisotropic relaxation of the retinal epithelium is visualized by progressive elongation of the hole in the glial sheet in a direction parallel to the retinal margin over several minutes (Fig. 6A-A”’ and Additional file 13: Movie S5). The directions of the principal strain axes typically deviate by 20 degrees or less from the orientation of the columns, and in only one case does the deviation exceed 35 degrees, making the identification of one of the principal strain axes as the one along the column direction unambiguous. Much of the variability in principal strain directions that does exist appears to be attributable to irregularities in the shapes of lesioned regions. These observations imply that glial processes support a substantial tensile mechanical stress along the cone columns.Additional file 13:
**Movie S5** Relaxation of retinal epithelium after glial ablation. Reconstruction of the Müller glial GFP reporter at the OLM surface from selective projections of live multiphoton confocal images from a double transgenic *ruby* zebrafish, with reporters for Müller glia (*gfap*:EGFP in green) and Red cones (*trß2*:tdTomato in red). The four frames in the movie (from Fig. 6A–A”’) show the OLM surface pre-ablation and at intervals of 9, 27, and 54 min, respectively, after targeted ablation of Müller glia. Photoreceptor profiles outside the ablation that were used to measure *x-* and *y-*strain are indicated by white dots. (AVI 12660 kb)


## Discussion

**Fig. 7:**
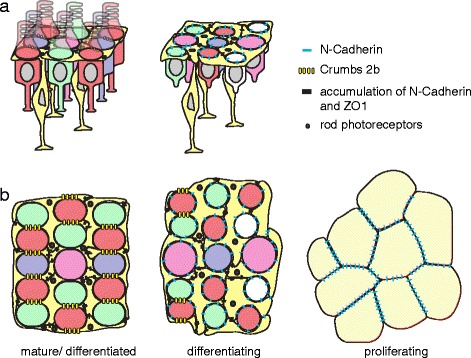
A model for how the multiplex photoreceptor lattice mosaic is patterned by anisotropic tension, glial scaffolding, and planar polarized cell adhesion. Retinal margin in **a** lateral and **b** planar views with expression and localization of cell-cell adhesion complexes (N-cadherin, Crb2b, and ZO-1) as photoreceptors and Müller glia progress from proliferating progenitors (right) to differentiating cells in the pre-column zone (center) to the fully patterned, mature cone lattice mosaic (left). Cones (red, green, blue with UV represented by magenta); rods (small black dots); Müller glia (bright yellow); retinal precursors (light yellow). See Discussion for additional details

Our major findings are summarized in Fig. 7. The apical profiles of neuroepithelial cells in the germinal zone are irregular polygons (Fig. 7, right), typical of proliferating epithelia [56, 57], with adherens junctions stabilized by N-cadherin and ZO-1 [55, 58, 59]. As newly generated photoreceptors begin to differentiate (Fig. 7, center), their apical profiles become rounder and smaller, and strongly labeled for activated phospho-Myosin II, consistent with apical constriction mediated by actomyosin contractility [54]. Parallel ridges of glial processes aligned with the retinal margin also become visible at this stage; the processes completely surround the photoreceptors, but form thicker, straighter, more prominent lines between the emerging single cell width columns of immature cones. In this pre-column zone, photoreceptor subtypes already display basic features of their final spatial pattern – a repetitive distribution and heterotypic correlations with the correct number of nearest neighbors of the correct (different) subtypes. However, the size of cone profiles at the level of the OLM and the spacing between adjacent cones is still quite variable, and organization into straight columns parallel to the margin is not yet apparent. Ordered, lattice packing, characterized by straight columns, narrow distributions of cone sizes and spacings, and planar-polarized localization of Crb, is attained only in the mature retina (Fig. 7, left).

Specific markers and distinctive morphological characteristics support the inference that correct spatial patterning of cone subtypes and rods is roughly coincident with onset of photoreceptor differentiation and precedes their packing into a precise lattice mosaic. The most compelling evidence comes from analysis of *trβ2*:tdTomato, which labels fate-committed, proliferative Red cone progenitors and mature Red cones [25]. As Red cones begin to differentiate in the pre-column zone, they are appropriately positioned with reference to the hexagonal pattern of mature Red cones in the adjacent lattice. Lineage tracing of *trβ2*: tdTomato+ progenitors in embryonic zebrafish retina showed that their terminal mitotic division produces two Red cones [25], suggesting that post-mitotic, immature Red cone daughter cells must move apart tangentially as they differentiate. Tangential migration is an important mechanism to establish homotypic mosaics [4], and tangential movement of horizontal cells has been observed with time-lapse imaging in embryonic zebrafish retina [60]. Local, homotypic repulsive interactions of developing dendrites contribute to evenly spaced mosaics of non-photoreceptor retinal neurons [4], and although photoreceptors do not have dendrites, they do possess ‘telodendria’, fine fibers radiating from the axon terminal [61] that mediate gap junction connections between cones [62]. A recent study of zebrafish embryonic retinas suggested that immature Red cones have telodendria [25], which could provide repulsive signals driving tangential migration. Future improvements and refinements in methods may allow for time-lapse imaging of the growing retina in juvenile zebrafish, which would help address these unanswered questions.

The question of when cone subtype cell fate is specified remains ambiguous, since specific markers for cone spectral subtypes are not available for immature, differentiating cones (with the exception of *trβ2* for Red cones). However, UV cones have distinctively large, round apical profiles, and similar profiles are present in the center of Red cone hexagons in the pre-column zone, at locations predicted by the row pattern of alternating Blue and UV cones in the adjacent lattice mosaic. The early appearance of these immature UV cones is intriguing, since *tbx2b* mutant zebrafish, in which UV cones largely fail to differentiate, have severe defects in the multiplex photoreceptor lattice mosaic pattern, even though other cone subtypes are produced in the correct ratio [14].

Additional indirect evidence supporting the inference that subtype identity of cone photoreceptors is likely established prior to column packing is that the ratio of *trβ2*: tdTomato+ immature Red cones to other immature cones and rods in the differentiating pre-column zone approximately matches the predicted ratio of 1:4 in the mature, multiplex lattice mosaic. Furthermore, the first contiguous single-cell wide column of cones to emerge from the pre-column zone contains the correct ratio of cone subtypes, with Red cones separated, alternately, by one and three immature cones. That the differentiating cones in the pre-column zone have established their correct identity is also supported by the planar polarized pattern of Crb2b immunoreactivity at the cell-cell interfaces between pentameric cone units (Green-Red-Blue-Red-Green) within a column [10, 27]. These pentameric Crb2b ‘ladders’, each separated by an intervening (Crb2b-negative) immature UV cone, are already apparent immediately peripheral to the first contiguous column of cones. The mechanisms through which cones acquire their spectral cell identity in zebrafish are not understood, and this study was not designed to probe that question. Almost certainly, the process of cone photoreceptor cell fate determination involves stepwise decisions driven by binary transcriptional switches regulating specific opsins and other features that distinguish cone subtypes in both vertebrate and invertebrate species, including mammals and fruit flies [63–65].

These studies suggest a possible, novel function for Müller glia as potential organizers of the multiplex photoreceptor lattice. This hypothesis derives from observations at three distinct stages, corresponding, respectively, to the pre-column zone, the transition to the mature lattice mosaic, and the mature lattice mosaic. In the mature lattice mosaic, apical glial processes establish a periodic scaffold parallel to the retinal margin preferentially localized between cone columns [10]. Unexpectedly, we found that apical processes of immature Müller glia throughout the pre-column zone are also distributed in periodic, linear bands parallel to the retinal margin that prefigure the cone columns. This is the earliest manifestation of a linear alignment, prior to evidence of single cell width cone columns.

Within the pre-column zone, differentiating photoreceptors show high levels of pMyoII, indicative of strong cortical constriction. N-cadherin and ZO-1 localize to homophilic glial junctions and adherens junctions between glia and differentiating cones, suggesting that forces generated by the actomyosin cortical network in photoreceptors and cadherin-mediated tension sensors likely mediate a gradual evolution in cell shape and position across the pre-column zone [54, 55, 66]. At the transition from pre-column zone to mature mosaic there is an abrupt reorganization of both adhesion molecules and pMyoII. In photoreceptors, N-cadherin expression is lost and levels of cortical pMyoII decrease, even as planar-polarized Crb-mediated adhesion appears between cones within columns. This change in the predominant photoreceptor adhesion molecules would favor a decrease in the length of cone-glial junctions and a corresponding increase in the length of cone-cone junctions, consistent with the observed retraction of glial processes into straighter bands and expansion of cone contacts within columns. Simultaneously, punctate adherens junctions colocalizing with high levels of pMyoII appear between glia, suggesting that their processes actively contract and straighten to corral cones into correct positions within the columns. These data point to dynamic remodeling of adhesion molecules and cytoskeleton as a potential driving force for cell shape and position changes that create the final, highly ordered packing at the transition from pre-column zone to mature multiplex mosaic. A similar scenario has been proposed for other, simpler epithelial systems [54, 66, 67].

In the mature mosaic, parallel bands of glia stabilized by N-cadherin-mediated adherens junctions alternate with columns of cones held by planar polarized Crb2b adhesion. The concentration of activated pMyoII in punctate junctions suggests that these glial bands remain under tension, forming a physical barrier between cone columns and contributing to mechanical stress anisotropies in the epithelial sheet, analogous to the role of the contractile actomyosin cables that separate cells at compartment boundaries in developing *Drosophila* wing [68] or that establish cell columns in *Drosophila* embryonic ventral epidermis [69, 70]. Remarkably, a function performed by the cortical cytoskeleton inside epithelial cells in *Drosophila* would thus, in the vertebrate retina, instead to be taken over by apical processes from glial cells in a different layer interposed between photoreceptors at the OLM.

We used laser ablation to directly demonstrate the anisotropic mechanical properties of Müller glia in the plane of the epithelium near the retinal margin. When the radial fiber of an individual Müller glia is targeted with laser ablation, its apical processes are lost from a confined region of the OLM, which then preferentially expands (recoils) in the direction parallel to the retinal margin. This indicates that, before ablation, the glial processes had exerted a tensile mechanical force parallel to the margin. Notably, almost all previous applications of laser ablation to probe tissue mechanics have either directly targeted particular junctional structures or made cuts across the epithelial surface on the scale of many cell diameters; here, in contrast, we took advantage of the retina’s unique architecture to remove selected glial cells and so to demonstrate a specific mechanical function for glial processes whose width can be as small as tens of nanometers [71]. Combined with the observation that glia cells form thick, phospho-Myosin-rich bands between cone columns, these results suggest that glia generate anisotropic mechanical tension across the retinal epithelium that could mediate formation of the photoreceptor lattice mosaic. Because the hexagonal pattern of Red cones was maintained after glial ablation, additional mechanical forces, such as Crb-mediated adhesive interactions between apical processes of rod and cone photoreceptors [27], must also contribute to stabilization and maintenance of the mature lattice mosaic pattern.

A direct test of the hypothesis that the anisotropic tensile forces generated by Müller glial processes not only stabilize the mature cone mosaic but also organize cones into parallel columnar arrays would be to remove Müller glia from the pre-column zone at the retinal margin and observe the effect on column formation. The laser ablation method we developed for targeting individual Müller glia is not useful for this experiment because the time window for live-imaging was limited to 1 h, and our estimate of retinal growth rates suggested that a new column forms over a period of ~ 8 h. Future studies incorporating more prolonged time-lapse imaging of juvenile zebrafish retina will require adapting techniques to provide sustained immobility and viability, likely including gill perfusion. Alternative methods for eliminating Müller glia might also be explored. A recent investigation in embryonic zebrafish used pharmacological inhibitors of the Notch signaling pathway (e.g.*,* DAPT) applied systemically and continuously in a narrowly defined developmental window beginning just prior to onset of Müller glial differentiation (45 h post-fertilization, hpf, through 96 hpf) to block their formation [34]. Embryonic zebrafish retinas that lacked Müller glia frequently split or ripped (a retinoschisis phenotype) and exhibited a decrease in resistance to tensile forces applied orthogonal to the surface at 72 and 96 hpf [34]. As these authors point out, the retina is not yet functional at these early embryonic stages, but once vision is established, Müller glia are crucial for function and survival of photoreceptors, so it is unlikely that retinas lacking Müller glia would develop to juvenile stages. We did attempt to block formation of Müller glia at the retinal margin by systemic treatment of juvenile zebrafish with DAPT, but these experiments were unsuccessful in preventing addition of new Müller glia at the growing retinal margin (data not shown).

Müller glia have been implicated in many different aspects of retinal biology, including neuronal development and differentiation, structural support of complex, laminated cytoarchitecture, regulation of metabolic and homeostatic functions, and modulation of neuronal activity [31]. Direct measurements of viscoelastic properties of isolated Müller glia revealed a soft, compliant mechanical substrate, which accommodates activity-dependent local swelling in synapses and facilitates neurite outgrowth [32, 72]. In retinal explants, the radial processes of Müller glia function as springs [34], and Müller glia respond to mechanical stretch orthogonal to the surface with increased calcium levels and changes in gene expression, suggesting that they act as radial tension sensors [33]. Recently, radial glia have also been implicated in shaping tissue-level deformations such as gyrification of the mammalian cerebral cortex [73]. In developing cerebellum, the related Bergmann glia establish anchoring centers that shape cerebellar foliation [74]. All of these examples primarily affect organization along the apical-basal dimension of the neural/retinal epithelium. The present study demonstrates a novel mechanical property of Müller glia: they mediate anisotropic mechanical forces in the planar dimension of the retina that are aligned with the retinal margin and the lattice mosaic array of cone photoreceptors.

## Conclusions

How developing nervous systems produce exquisitely ordered spatial arrangements of neurons is a longstanding but unanswered puzzle. The periodic, crystalline lattice of cone photoreceptors in fish retinas is a striking example of a highly orderly cell arrangement. In earlier work, we developed a mathematical model of cell packing, which predicted that anisotropic mechanical tension in the retinal epithelium orients planar polarized adhesive interfaces to align the mosaic pattern. In this study we developed a robust technique for live-imaging of photoreceptors and Müller glia in juvenile zebrafish with cell-specific fluorescent markers, providing the first in vivo view of how the crystalline mosaic pattern of photoreceptors is created. Unexpectedly, we found that parallel bands of Müller glial stabilized by N-cadherin-mediated adherens junctions, which separate cone columns in the mature mosaic, appear prior to columnar packing of developing cones at the growing retinal margin. Accumulation of phosphorylated MyosinII at punctate adherens junctions between Müller glial processes indicates that these glial bands are under tension. We then used targeted laser ablation to probe tissue mechanics. These experiments directly demonstrated that Müller glial processes generate mechanical stress anisotropies in the epithelial retinal sheet as predicted. Although Müller glial cells are known to provide mechanical and physiological support to the retina, this is the first demonstration, to our knowledge, of planar epithelial forces mediated by glia that are aligned with planar spatial patterning of vertebrate neurons.

Recent studies with stem and progenitor cells have highlighted the remarkable self-organizing properties of developing tissues and the biomechanical mechanisms that regulate cell shape and position. These studies have largely employed embryonic or larval systems, notably *Drosophila* but also other invertebrate or vertebrate animal models, including zebrafish embryos. In these cases, the epithelium investigated was at or near the surface of the tissue. Our study is unique in that we examined formation of the highly regular crystalline lattice of photoreceptor neurons and glial cells within an epithelial layer located deep in the living fish eye; we were thus able to directly examine pattern formation at later stages of development that are usually inaccessible to live imaging.

## Additional files

Additional file 1: Table S1. Antibody List. (DOCX 65 kb)

Additional file 2: Figure S1. Maximum intensity *z-*projection and lateral slice view of retinal margin illustrating shape and position of mitotic figures. (PDF 1495 kb)

Additional file 3: Movie S1. 3D-reconstruction of the peripheral retina and overlying retinal pigmented epithelium demonstrates the close apposition of these two epithelia at the retinal germinal zone. Maximum intensity *z-*projection of the retinal margin in a flat-mount preparation of neural retina and overlying retinal pigmented epithelium, immunostained for ZO1 (green). (See also Fig. 1G.) (AVI 3892 kb)

Additional file 4: Movie S2. Live imaging of the photoreceptor mosaic emerging at the retinal margin in rapidly growing juvenile zebrafish. Confocal *z*-stack series of live multiphoton confocal imaging at the dorsal retinal margin in a double transgenic *ruby* zebrafish; *Tg*(*trß2*:tdTomato) in red and *Tg*(*crx*:mCFP) in cyan. The *crx* promoter is expressed in all cone and rod photoreceptors; the mCFP reporter localizes to the plasma membrane. The tdTomato+ proliferative retinal progenitors are randomly distributed in the most peripheral region of the germinal zone (asterisks). The first cone column (arrow and inset) is composed of tdTomato+ Red cones alternately separated by mCFP-labeled profiles of one or three immature cones (white dots), as predicted by the organization of cone types in a column in the mature mosaic. Mature cones develop long apical projections, including conical-shaped outer segments that are strongly labeled by the *crx*:mCFP reporter. (See also Fig. 2D.) (AVI 1851 kb)

Additional file 5: Figure S2. Immature UV cones have rounded apical profiles in the pre-column area. (PDF 939 kb)

Additional file 6: Figure S3. Rod photoreceptors are incorporated into the cone mosaic as it emerges from the proliferating zone. (PDF 146 kb)

Additional file 7: Figure S4. Segmentation and classification of photoreceptor profiles. (PDF 1146 kb)

Additional file 8: Figure S5 Müller glial apical processes are preferentially distributed into parallel, inter-column bands. (PDF 1085 kb)

Additional file 9: Movie S3. Müller glial apical processes provide scaffolding for differentiating photoreceptor cells. 3D–reconstruction (maximum intensity *z-*projection) of live multiphoton confocal imaging from a double transgenic *ruby* zebrafish, with reporters for Müller glia (*gfap*:EGFP in green) and Red cones (*trß2*:tdTomato in red). Müller glial processes extend laterally at the level of OLM to surround profiles of individual Red cones and other photoreceptors. (See also Fig. 4A–D.) (AVI 4930 kb)

Additional file 10: Movie S4. Red cones in the pre-column zone (magenta dots) and in the mature mosaic (white dots) are surrounded by Müller glial scaffolding. Higher magnification of a portion of the field shown in Fig. 4A–D and Additional file 9: Movie S3: Müller glia (green) and Red cones (red). Weak GFP signals of immature Müller glia first appear in the proliferative zone (toward the right). The intensity of the GFP signal increases in differentiating Müller glia and their processes surround photoreceptors, including differentiating Red cones (magenta dots) at the level of the OLM. Emergence of the hexagonal distribution of Red cones (white dots) is accompanied by morphological maturation of Müller glia. (AVI 4600 kb)

Additional file 11: Figure S6. Expression of *cdh2* transcripts in differentiating photoreceptors (A-A”’) In situ hybridization for *cdh2* transcripts (A, white or A”, A”’, magenta) in a retinal cross-section from the Müller glial reporter line, *Tg*(*gfap*:EGFP) (A’-A”’, green). (PDF 2467 kb)

Additional file 12: Figure S7. 3D reconstruction demonstrates complete ablation of targeted Müller glia near the retinal margin. (PDF 1863 kb)

## Abbreviations

cdh2: N-cadherin
Crb: Crumbs
crx: Cone rod homeobox
D: Dorsal
EdU: 5-ethynyl-2’-deoxyuridine
EGFP: Enhanced green fluorescent protein
gcl: Ganglion cell layer
gfap: Glial fibrillary acidic protein
hpf: Hours post-fertilization
Inl: Inner nuclear layer
MP: Multiphoton
N: Nasal
OLM: Outer limiting membrane
onl: Outer nuclear layer
PB: Phosphate buffer
PBS: Phosphate buffered saline
pH3: Phospho-Histone H3
pMyoII: Phosphorylated Myosin II
rh1 (formerly, rho): Rhodopsin
sws1: Short-wavelength-sensitive opsin 1
sws2: Short-wavelength-sensitive opsin 2
T: Temporal
tr*β*2: Thyroid receptor beta
V: Ventral
ZO1: Zonula Occludens 1
