# Supplementary material for: Anisotropic Müller glial scaffolding supports a multiplex lattice mosaic of photoreceptors in zebrafish retina
Source: Neural Dev. 2017 Nov 15;12:20. doi: 10.1186/s13064-017-0096-z (PMC5688757; doi:10.1186/s13064-017-0096-z)
Supplement: Supplementary file 2 — Antibody List. (DOCX 65 kb) [file 13064_2017_96_MOESM1_ESM.docx]

**Table S1 Antibody List**

| **antibody description** | **source** | **dilution** |
| --- | --- | --- |
| mouse anti-Zonula Occludens | Invitrogen | 1:200 |
| rabbit anti-Phospho Histone 3 | Cell Signaling Technology | 1:200 |
| rabbit anti-dsRed | Clontech | 1:200 |
| rabbit anti-GFP | Invitrogen | 1:500 |
| mouse anti-ZS5 (Crb2a) | Zebrafish International Resource Center | 1:200 |
| rabbit anti-Crb2b | gifted from Xiangyun Wei | 1:200 |
| rabbit anti-N-Cadherin | Abcam | 1:200 |
| mouse anti-PanCadherin | Abcam | 1:200 |
| rabbit anti-phospho-Myosin Light Chain 2 (Ser19) | Cell Signaling Technology | 1:200 |
| anti-mouse IgG DyLight549 | Jackson ImmunoResearch | 1:400 |
| anti-mouse IgG DyLight549 | Thermo Fisher | 1:400 |
| anti-rabbit IgG DyLight647 | Jackson ImmunoResearch | 1:400 |
| anti-rabbit IgG DyLight647 | Thermo Fisher | 1:400 |
