# Supplementary material for: Anisotropic Müller glial scaffolding supports a multiplex lattice mosaic of photoreceptors in zebrafish retina
Source: Neural Dev. 2017 Nov 15;12:20. doi: 10.1186/s13064-017-0096-z (PMC5688757; doi:10.1186/s13064-017-0096-z)
Supplement: Supplementary file 3 — Maximum intensity z-projection and lateral slice view of retinal margin illustrating shape and position of mitotic figures. (PDF 1495 kb) [file 13064_2017_96_MOESM2_ESM.pdf]

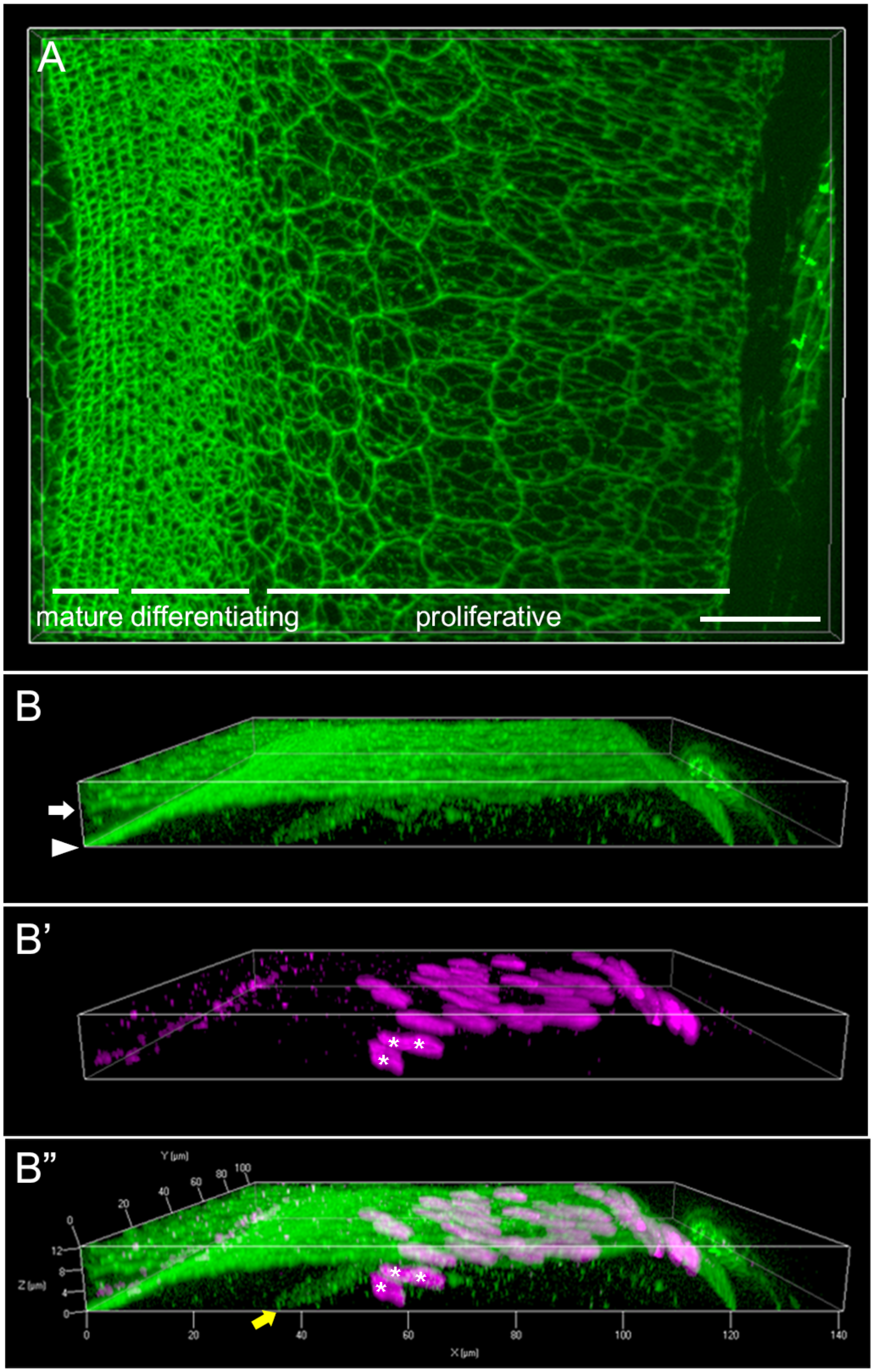

Figure S1

Figure S1. Maximum intensity *z*-projection and lateral slice view of retinal margin illustrating shape and position of mitotic figures. (A) Snap shot from a 3D-

reconstruction of the retinal margin and retinal pigmented epithelium (RPE) in a flat-mount preparation immunostained for ZO1 (green). The front of the eye is to the right. Proliferative, differentiating (pre-column), and differentiated (mature mosaic) zones of the retina are labeled. (B-B”) Lateral view (*xz* plane) of ZO1 (green) and the mitotic marker, pH3 (magenta). (B) The RPE (arrow) and neural retinal epithelium (arrowhead) are separate but closely apposed, especially in the proliferative zone. (B’) The pH3+ mitotic cells are tilted with respect to the surface of the retinal epithelium, and the resultant parallax precludes identifying the profiles of pH3+ mitotic cells in the ZO1 channel in a maximum intensity *z*-projection (*e.g.,* Fig. 1G'). (B’’) Proliferating endothelial cells in the circumferential blood vessel (yellow arrow) of the vitreous circulation, which lies below the retinal germinal zone [1], are also pH3+ (asterisks). Scale bar: 20 µm (A).

1. Raymond PA, Barthel LK, Bernardos RL, Perkowski JJ. Molecular characterization of retinal stem cells and their niches in adult zebrafish. BMC Dev Biol. 2006;6:36. http://dx.doi.org/10.1186/1471-213X-6-36
