# Supplementary material for: Anisotropic Müller glial scaffolding supports a multiplex lattice mosaic of photoreceptors in zebrafish retina
Source: Neural Dev. 2017 Nov 15;12:20. doi: 10.1186/s13064-017-0096-z (PMC5688757; doi:10.1186/s13064-017-0096-z)
Supplement: Supplementary file 6 — Immature UV cones have rounded apical profiles in the pre-column area. (PDF 939 kb) [file 13064_2017_96_MOESM5_ESM.pdf]

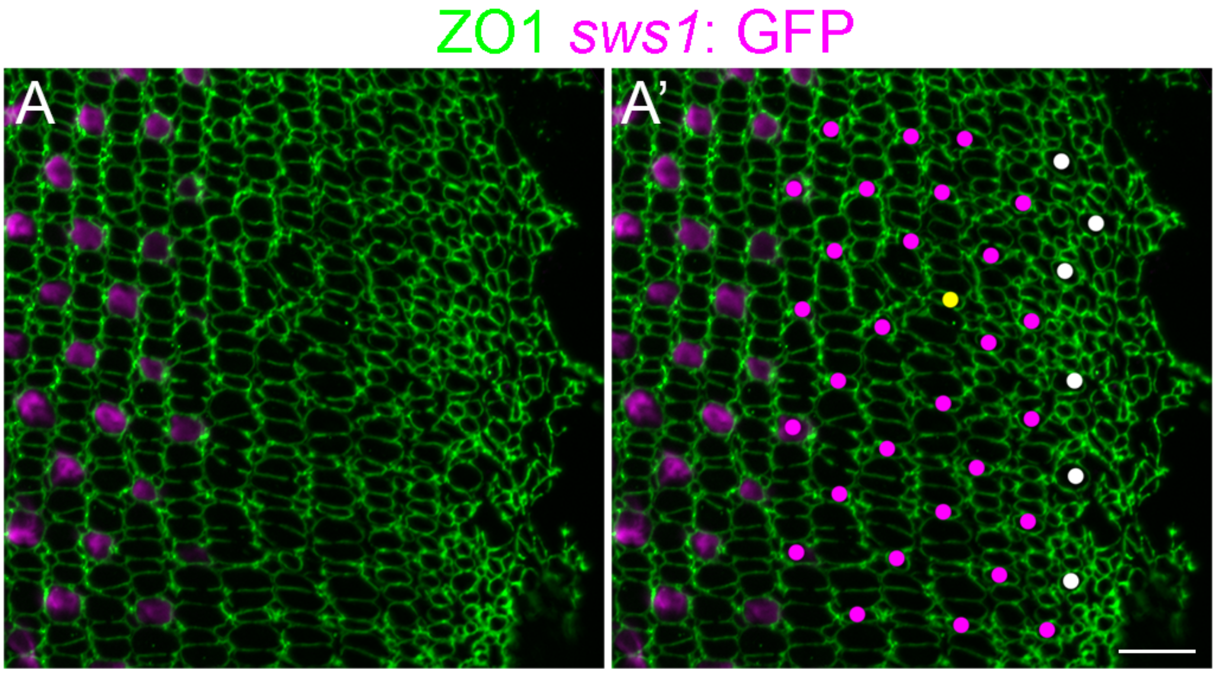

Figure S2

Figure S2. Immature UV cones have rounded apical profiles in the pre-column area. (A, A') Retinal flat-mount immunocytochemistry for ZO1 (green) in a juvenile transgenic zebrafish with the UV cone reporter, *Tg*(*sws1*: EGFP) (magenta). The onset of *sws1* opsin transgene expression is delayed in the differentiating UV cones until after the organization of cone columns. (A') The precision of the mosaic pattern allows profiles that are *Tg*(*sws1*: EGFP)-negative to be identified as UV cones (magenta dots), and the position and shape of the large rounded profiles in the pre-column zone (white dots) suggests that these, too, are differentiating UV cones. Note the appearance of a new row and column (yellow dot) in this region, a pattern defect called a 'Y-junction' [1], which is analogous to an edge dislocation in crystals grown on curved surfaces and is required to accommodate the increased perimeter with continued retinal growth. Scale bar: 10 μm.

1. Nishiwaki Y, Oishi T, Tokunaga F, Morita T. Three-dimensional reconstitution of cone arrangement on the spherical surface of the retina in the medaka eyes. Zool Sci. 1997;14(5):795-801. http://dx.doi.org/10.2108/zsj.14.795
