# Supplementary material for: Anisotropic Müller glial scaffolding supports a multiplex lattice mosaic of photoreceptors in zebrafish retina
Source: Neural Dev. 2017 Nov 15;12:20. doi: 10.1186/s13064-017-0096-z (PMC5688757; doi:10.1186/s13064-017-0096-z)
Supplement: Supplementary file 7 — Rod photoreceptors are incorporated into the cone mosaic as it emerges from the proliferating zone. (PDF 146 kb) [file 13064_2017_96_MOESM6_ESM.pdf]

A

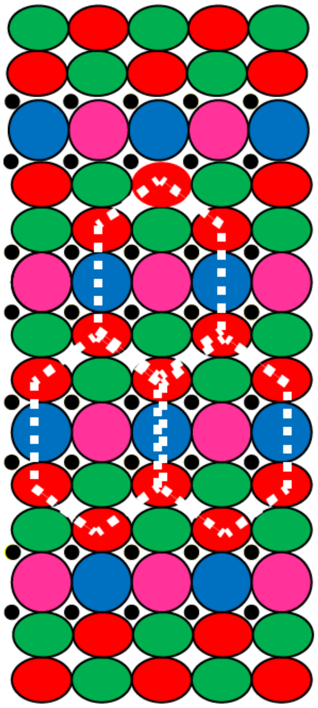

B

	trβ2+	*trβ2-*			
	R	G	B	UV	rod
# in a hexagon	6	2	2	1	4
# hexagons sharing	3	1	2	1	1
total / hexagon	2	2	1	1	4
predicted cell ratios	1	2			
	1	4			

C

	trβ2+	*trβ2-*	ratio *trβ2+*/*trβ2-*
Retina 1	72	302	4.19
Retina 2	62	344	5.55
**Total**	**134**	**646**	**4.87**

Figure S3

Figure S3. Rod photoreceptors are incorporated into the cone mosaic as it emerges from the proliferating zone. (A) Schematic of photoreceptor mosaic with red, green, blue, and magenta (UV) cones (large circles) and rods (small black circles). Hexagonal pattern of Red cones (white dotted lines). (B) Expected count of Red (R), Green (G), Blue (B), and UV cones contributing to each hexagon, cones sharing adjacent hexagons, the number (#) per hexagon, and the expected ratios of *trβ2+* (Red) to *trβ2-* (Green+Blue+UV) photoreceptor profiles (with and without rods) in flat-mounts of *trβ2*:tdTomato retinas immunostained for ZO1 (*e.g.,* Fig. 2E). (C) Results from automated cell counting analysis of two retinas (n=780 total cells counted). (See also Fig. S4.)
