# Supplementary material for: Anisotropic Müller glial scaffolding supports a multiplex lattice mosaic of photoreceptors in zebrafish retina
Source: Neural Dev. 2017 Nov 15;12:20. doi: 10.1186/s13064-017-0096-z (PMC5688757; doi:10.1186/s13064-017-0096-z)
Supplement: Supplementary file 8 — Segmentation and classification of photoreceptor profiles. (PDF 1146 kb) [file 13064_2017_96_MOESM7_ESM.pdf]

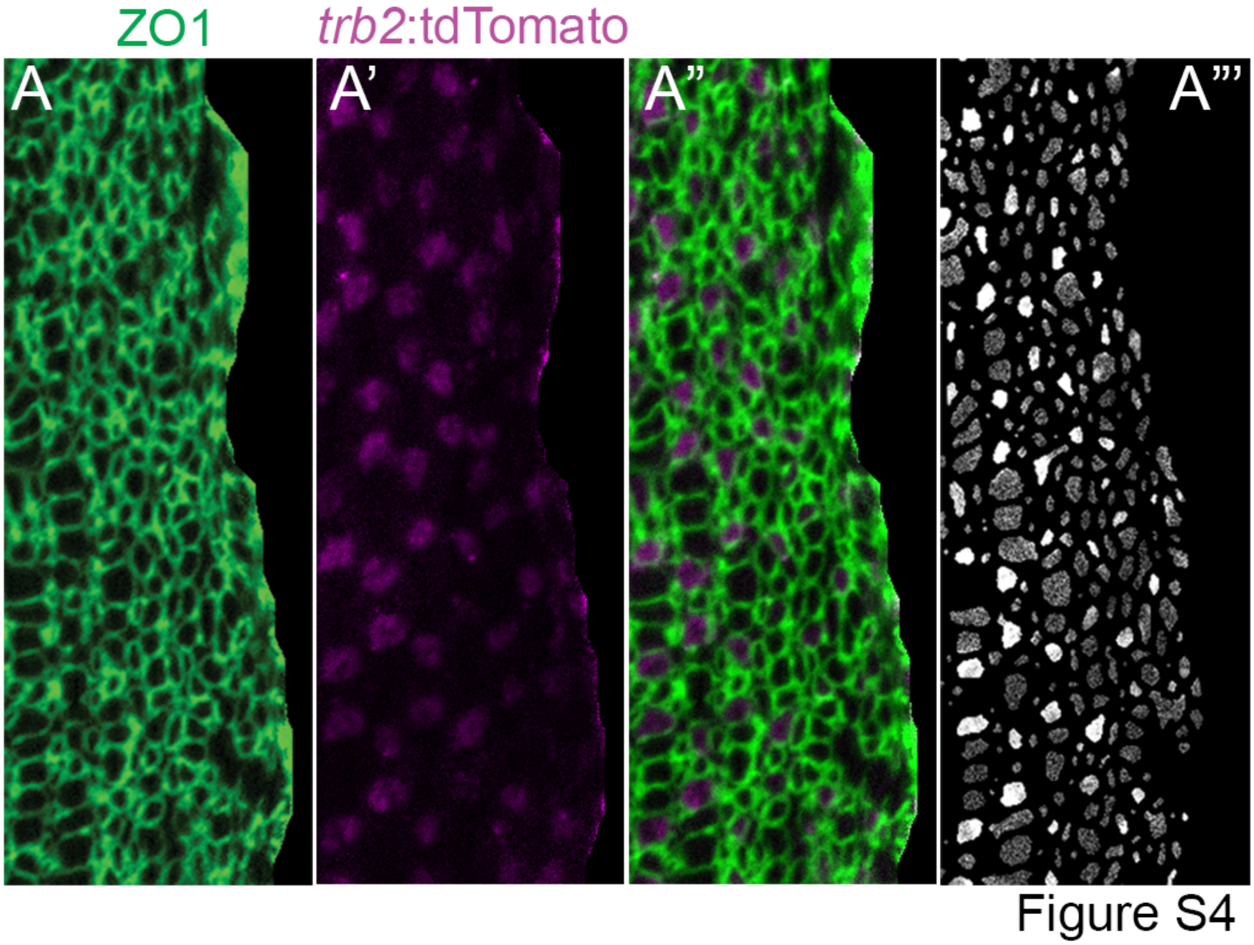

Figure S4. Segmentation and classification of photoreceptor profiles. Selective projection of ZO1-labled cell profiles (A, A'') and *trβ2*:tdTomato+ Red cones (A', A'') in the pre-column zone of a retinal flat-mount. Panel A''' shows the tdTomato signal intensity at the level of the segmented ZO-1-labeled cell profiles, which was used for the automated classification and counting procedure described in the Materials and Methods.
