# Supplementary material for: Anisotropic Müller glial scaffolding supports a multiplex lattice mosaic of photoreceptors in zebrafish retina
Source: Neural Dev. 2017 Nov 15;12:20. doi: 10.1186/s13064-017-0096-z (PMC5688757; doi:10.1186/s13064-017-0096-z)
Supplement: Supplementary file 9 — Müller glial apical processes are preferentially distributed into parallel, inter-column bands. (PDF 1085 kb) [file 13064_2017_96_MOESM8_ESM.pdf]

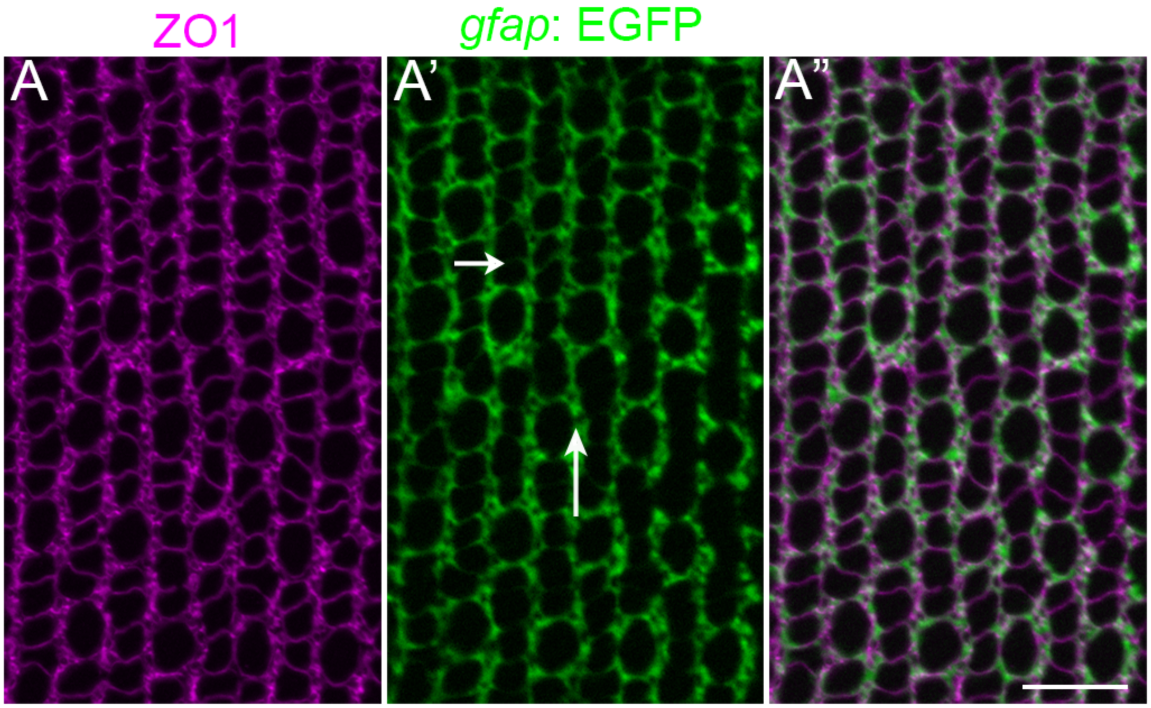

Figure S5

Figure S5. Müller glial apical processes are preferentially distributed into parallel, inter-column bands. (A-A”) Retinal flat-mount immunocytochemistry for ZO1 (magenta) in the Müller glial transgenic reporter line, *Tg*(*gfap*: EGFP). Müller glial processes (GFP+) completely surround the profiles of individual rods and cones, forming relatively thin intra-column lamellae (short arrow), and thicker inter-column expansions (long arrow). Refer to Fig 1 A and B for a description of columns. Scale bar: 10 μm.
