# Supplementary material for: Anisotropic Müller glial scaffolding supports a multiplex lattice mosaic of photoreceptors in zebrafish retina
Source: Neural Dev. 2017 Nov 15;12:20. doi: 10.1186/s13064-017-0096-z (PMC5688757; doi:10.1186/s13064-017-0096-z)
Supplement: Supplementary file 12 — Expression of cdh2 transcripts in differentiating photoreceptors (A-A”’) In situ hybridization for cdh2 transcripts (A, white or A”, A”’, magenta) in a retinal cross-section from the Müller glial reporter line, Tg(gfap:EGFP) (A’-A”’, green). (PDF 2467 kb) [file 13064_2017_96_MOESM11_ESM.pdf]

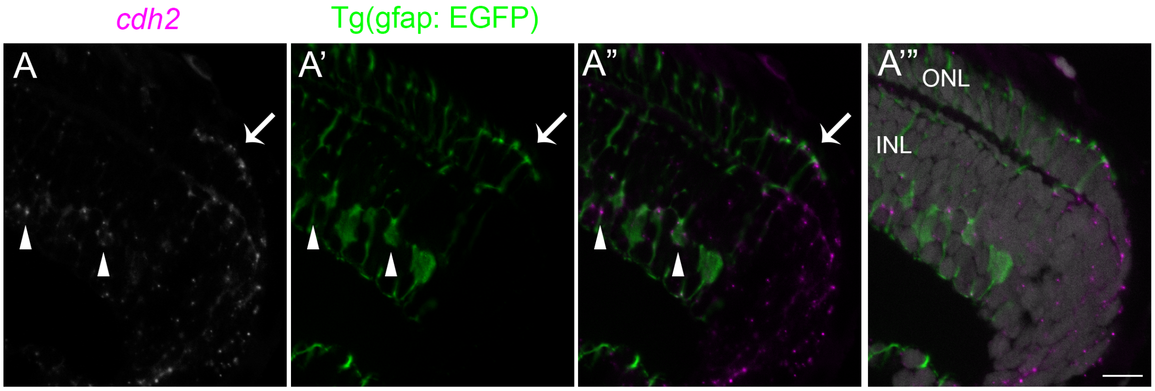


Figure S6

**Figure S6. Expression of *cdh2* transcripts in differentiating photoreceptors**

(A-A''') *In situ* hybridization for *cdh2* transcripts (A, white or A'', A''', magenta) in a retinal cross-section from the Müller glial reporter line, *Tg*(*gfap*:EGFP) (A'-A''', green). *In situ* hybridization was performed as previously described [1]. In brief, after rehydration and proteinase K treatment, retinal cross-sections were incubated with digoxigenin-labeled RNA probes for *cdh2* in Hauptmann's hybridization solution at 65C overnight. Sections were then washed, treated with alkaline phosphatase conjugated anti-digoxigenin antibody (Roche, Basel, Switzerland), and blocked with Maleate Blocking solution. The signal was detected with Fast Red (Roche, Basel, Switzerland). Proliferating progenitors in the germinal zone (on the right), immature cone photoreceptors (arrow), and mature Müller glia (arrowheads) in the inner nuclear layer all express *cdh2* transcripts. Mature photoreceptors in the outer nuclear layer of the mature, differentiated retina (to the left) do not express *cdh2*. ONL, outer nuclear layer; INL, inner nuclear layer. Scale bar: 10 µm.


1. Raymond PA, Barthel LK, Bernardos RL, Perkowski JJ. Molecular characterization of retinal stem cells and their niches in adult zebrafish. BMC Dev Biol. 2006;6:36. http://dx.doi.org/10.1186/1471-213X-6-36
