# Supplementary material for: Anisotropic Müller glial scaffolding supports a multiplex lattice mosaic of photoreceptors in zebrafish retina
Source: Neural Dev. 2017 Nov 15;12:20. doi: 10.1186/s13064-017-0096-z (PMC5688757; doi:10.1186/s13064-017-0096-z)
Supplement: Supplementary file 13 — 3D reconstruction demonstrates complete ablation of targeted Müller glia near the retinal margin. (PDF 1863 kb) [file 13064_2017_96_MOESM12_ESM.pdf]

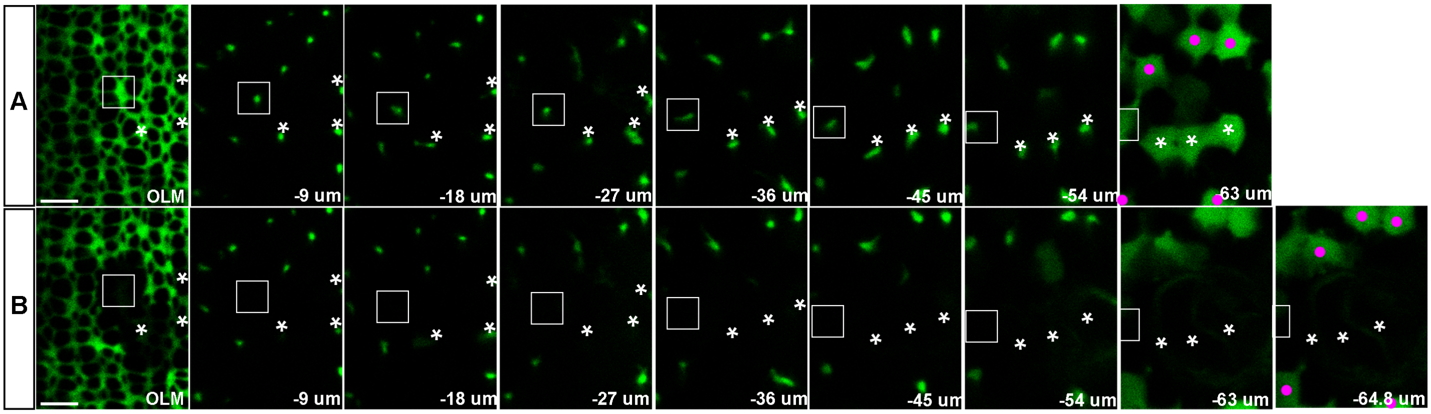

Figure S7. 3D reconstruction demonstrates complete ablation of targeted Müller glia near the retinal margin. Confocal *z*-stack series of *Tg*(*gfap*: EGFP)-labeled Müller glial processes from the level of the outer limiting membrane (OLM) to the cell body before (A) and 9 minutes after laser ablation (B). Multi-photon laser ablation targeted the radial processes at a depth of 25-50 μm, between the OLM and the level of the cell bodies (~63 μm below the OLM in this example). Three targeted Müller glial cell bodies and radial processes are indicated by asterisks. (B) Multi-photon laser ablation targeting the radial glial process at a single *z*-level successfully destroyed the cell body and ascending processes of Müller glia (asterisks) and created a hole in the sheet of Müller glial processes at the level of OLM. The surrounding Müller glia remained intact (magenta dots), except for one cell close to the cluster of three targeted cells, which disappeared after the ablation (rectangle). The slight difference in the relative distance from OLM to the cell bodies of surviving Müller glia before and after ablation (-63 μm and -64.8 μm, respectively) is consistent with a previous report that radial processes of Müller glial in embryonic zebrafish retinas function like a spring, providing tension in the plane perpendicular to the apical surface [1]. Scale bar: 5 μm (A, B).

1. MacDonald RB, Randlett O, Oswald J, Yoshimatsu T, Franze K, Harris WA. Muller glia provide essential tensile strength to the developing retina. Journal Cell Biol. 2015;210(7):1075-83. http://dx.doi.org/10.1083/jcb.201503115
